# Mutations in TRAF3IP1/IFT54 reveal a new role for IFT proteins in microtubule stabilization

**DOI:** 10.1038/ncomms9666

**Published:** 2015-10-21

**Authors:** Albane A. Bizet, Anita Becker-Heck, Rebecca Ryan, Kristina Weber, Emilie Filhol, Pauline Krug, Jan Halbritter, Marion Delous, Marie-Christine Lasbennes, Bolan Linghu, Edward J. Oakeley, Mohammed Zarhrate, Patrick Nitschké, Meriem Garfa-Traore, Fabrizio Serluca, Fan Yang, Tewis Bouwmeester, Lucile Pinson, Elisabeth Cassuto, Philippe Dubot, Neveen A. Soliman Elshakhs, José A. Sahel, Rémi Salomon, Iain A. Drummond, Marie-Claire Gubler, Corinne Antignac, Salahdine Chibout, Joseph D. Szustakowski, Friedhelm Hildebrandt, Esben Lorentzen, Andreas W. Sailer, Alexandre Benmerah, Pierre Saint-Mezard, Sophie Saunier

**Affiliations:** 1 Inserm UMR-1163, Laboratory of Hereditary Kidney Diseases, 75015 Paris, France; 2 Paris Descartes Sorbonne Paris Cité University, Imagine Institute, 75015 Paris, France; 3 Novartis Institutes for Biomedical Research, Basel CH-4002, Switzerland; 4 Department of Structural Cell Biology, Max-Planck-Institute of Biochemistry, 82152 Martinsried, Germany; 5 Division of Nephrology, Department of Medicine, Boston Children’s Hospital and Harvard Medical School, Boston, Massachusetts 02115, USA; 6 Division of Nephrology, Department of Internal Medicine, University Clinic Leipzig, 04103 Leipzig, Germany; 7 Novartis Institutes for Biomedical Research, Cambridge, Massachusetts 02139, USA; 8 Inserm UMR-1163, Genomic Core Facility, 75015 Paris, France; 9 Paris Descartes Sorbonne Paris Cité University, Bioinformatics Core Facility, 75015 Paris, France; 10 Cell Imaging Platform, INSERM US24 Structure Fédérative de recherche Necker, Paris Descartes Sorbonne Paris Cité University, 75015 Paris, France; 11 Department of Medical Genetic, Arnaud de Villeneuve University Health Center, 34090 Montpellier, France; 12 Nephrology department, L'Archet II Hospital, Nice University Health Center, 06202 Nice, France; 13 Hemodialysis-Nephrology Department, William Morey Hospital, 71321 Chalon-sur-Saône, France; 14 Department of Pediatrics, Center of Pediatric Nephrology and Transplantation, Cairo University, Egyptian Group for Orphan Renal Diseases, 11956 Cairo, Egypt; 15 INSERM U968, CNRS UMR 7210; Sorbonne Universités, Université Pierre et Marie Curie, UMR S968, Institut de la vision, 75012 Paris, France; 16 Centre Hospitalier National d'Ophtalmologie des Quinze-Vingts, INSERM, Direction de l'Hospitalisation et de l'Organisation des Soins, Centre d'Investigation Clinique 1423, 75012 Paris, France; 17 Assistance Publique—Hôpitaux de Paris, Pediatric Nephrologic department, Necker-Enfants Malades Hospital, 75015 Paris, France; 18 Nephrology Division, Massachusetts General Hospital, Charlestown, Massachusetts 02114, USA; 19 Department of Genetics, Harvard Medical School, Boston, Massachusetts 02114, USA; 20 Assistance Publique-Hôpitaux de Paris, Department of Genetics, Necker-Enfants Malades Hospital, 75015 Paris, France

## Abstract

Ciliopathies are a large group of clinically and genetically heterogeneous disorders caused by defects in primary cilia. Here we identified mutations in *TRAF3IP1* (TNF Receptor-Associated Factor Interacting Protein 1) in eight patients from five families with nephronophthisis (NPH) and retinal degeneration, two of the most common manifestations of ciliopathies. *TRAF3IP1* encodes IFT54, a subunit of the IFT-B complex required for ciliogenesis. The identified mutations result in mild ciliary defects in patients but also reveal an unexpected role of IFT54 as a negative regulator of microtubule stability via MAP4 (microtubule-associated protein 4). Microtubule defects are associated with altered epithelialization/polarity in renal cells and with pronephric cysts and microphthalmia in zebrafish embryos. Our findings highlight the regulation of cytoplasmic microtubule dynamics as a role of the IFT54 protein beyond the cilium, contributing to the development of NPH-related ciliopathies.

## Abstract (web summary)

Nephrophthisis (NPH) is a common manifestation of ciliopathy diseases. Here the authors identify mutations in intraflagellar transport 54 (IFT54) in patients with NPH and discover an extra-ciliary role for IFT54 in regulating cytoplasmic microtubule dynamics, that contributes to the pathophysiology of this disease.

## Graphical abstract

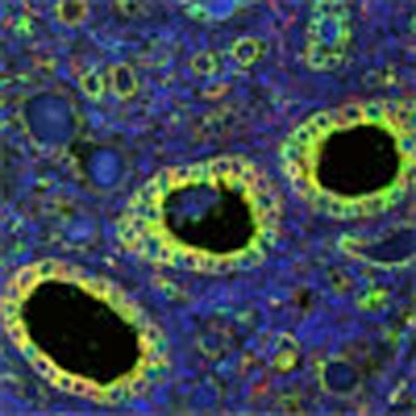

Nephronophthisis (NPH) is an autosomal-recessive nephropathy characterized by massive interstitial fibrosis, tubular basement membrane thickening and cyst formation, leading to end-stage renal disease (ESRD) during childhood^1^. NPH is a major manifestation of ciliopathies, a large group of diseases caused by dysfunction of the primary cilium^2^. The cilium is a microtubule-based organelle present at the surface of almost all vertebrate cells, which senses flow changes and mediates signalling pathways essential during development and tissue homeostasis, such as Hedgehog, Wnt/PCP and cAMP/PKA signalling. Intraflagellar transport (IFT) selects cargos at the base of the cilium and transports axonemal components required for cilia assembly, and proteins involved in ciliary signalling. The IFT-B complex, which consists of 16 different proteins, mediates anterograde transport by associating with kinesin II and has been shown to be essential for cilium formation^3^,^4^. Retrograde transport is mediated by dynein 2 and the 6 subunits of the IFT-A complex, however, inactivation of most IFT-A subunits does not lead to major defects in ciliogenesis^3^,^5^.

NPH and associated syndromes are clinically and genetically heterogeneous diseases. To date, NPH-causing mutations have been identified in more than 20 genes (*NPHP1–19;IFT140*)^6^,^7^,^8^, accounting for about 50% of all cases presenting with NPH^9^. Most of the NPHP-encoded proteins participate in ciliary function, either at the transition zone or as components of the IFT complex. All 6 IFT-A subunit encoding genes are frequently mutated in syndromic NPH, whereas mutations in only one IFT-B encoding gene, *IFT172*, have been detected so far^8^,^10^.

*TRAF3IP1* encodes the IFT-B subunit, IFT54, and its inactivation is embryonic lethal and causes characteristic ciliopathy phenotypes, including neural developmental defects, polydactyly and microphthalmia in mice^11^, and curved body axis, pronephric cysts and retinal degeneration in the *elipsa* zebrafish mutant^12^,^13^.

Our study demonstrates that hypomorphic mutations in the IFT-B protein IFT54 cause NPH with extrarenal defects. We linked these mutations with mechanistic features previously reported for other ciliopathies, including decreased ciliary cAMP signalling, hyperacetylation of cytoplasmic microtubules and defects in the establishment of cell junctions and polarity. Most importantly, this work describes an extra-ciliary role of the IFT54 protein in the regulation of cytoplasmic microtubule dynamics by modulating expression of MAP4. These data highlight a putative new mechanism responsible for NPH and associated phenotypes.

## Results

### Identification of *TRAF3IP1* mutations in NPH patients

Linkage analysis combined with whole-exome sequencing (WES) in parallel to targeted exome sequencing (‘ciliome’)^6^,^10^,^14^ conducted in 1,427 individuals with NPH revealed mutations in *TRAF3IP1* in eight individuals from five unrelated families (Table 1 and Supplementary Fig. 1a–g and Supplementary Tables 1 and 2). Three families carried three different homozygous missense mutations, whereas in one family, the affected individual NPH683–21 was compound heterozygous for a missense and a stop codon mutation (Table 1 and Supplementary Fig. 1a–d). Last, we identified a homozygous mutation in individual NPH1110-22 that creates a new donor splice site after exon 13, leading to a premature stop codon causing mRNA decay (Supplementary Figs 1e and 2a–d). All missense mutations were predicted to be damaging by Polyphen2, SIFT and/or PHRED2 (Table 1). Segregation of *TRAF3IP1* mutations with the disease was confirmed by Sanger sequencing in all families (Supplementary Fig. 1a–e).

**Figure 1:**
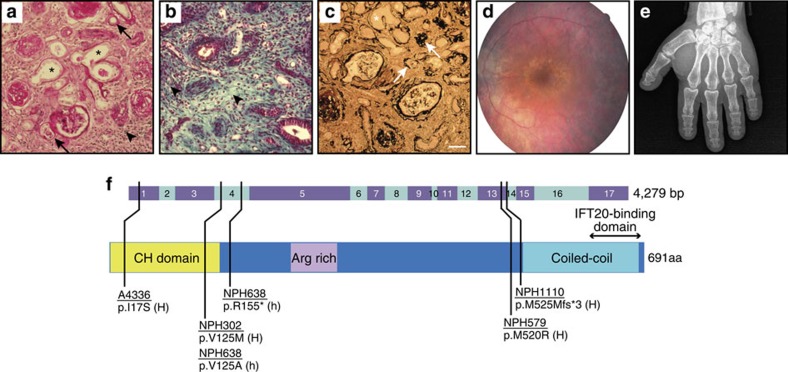
Identification of *TRAF3IP1* mutations in patients with nephronophthisis and retinal degeneration. Periodic acid schiff (**a**), trichrome (**b**) and silver methenamine (**c**) staining on kidney sections from individual NPH302-23 (**a**,**b**) and NPH1110-22 (**c**) revealed massive interstitial fibrosis (arrow heads) with cell infiltration, atrophic tubules with thickening of the basement membrane (arrows), as well as dilatation of proximal tubules (asterisks), characteristic of NPH. Scale bar, 50 μm. (**d**) Fundus photograph of individual NPH1110-22 showed characteristic aspects of RP, with pigmentary reorganization, papillary pallor and thin retinal vessels. (**e**) Left hand X-ray of individual NPH1110-22 showing short fingers (brachydactyly). (**f**) Organization of exons of *TRAF3IP1* cDNA (top panel) and functional domains of IFT54 protein with an N-terminal calponin homology (CH) domain involved in tubulin binding, an Arginine-rich motif and a C-terminal coiled-coil domain involved in IFT20 binding. Black bars indicate positions of the identified mutations. Family numbers are underlined. H, homozygous; h, heterozygous.

All of these patients presented with tubulointerstitial nephritis characteristic of NPH (leading to ESRD between 3 and 16 years). Microscopic analyses of kidney sections revealed massive interstitial fibrosis with inflammatory cell infiltration, atrophic tubules with thickening of the basement membrane, dedifferentiated tubules as well as dilatation of proximal tubules (Table 1, Fig. 1a–c). Consistent with Senior–Løken Syndrome, all patients with renal manifestation also developed early to late onset retinal dystrophy (between 2 months and 20 years) with infrequent occurrences of macular degeneration, nystagmus and strabismus (Table 1; Fig. 1d; Supplementary Fig. 3). Four patients also presented with liver defects (cholestasis, hepatic fibrosis or Caroli disease) and six with skeletal anomalies (polydactyly, microdactyly, small femoral heads; Table 1; Fig. 1e). Two patients from family NPH579 presented with clinical features of Bardet–Biedl Syndrome, i.e., developmental delay, retinal degeneration, polydactyly, obesity and hypogonadism (Table 1). These multisystemic defects are reminiscent of mutations in the *IFT172* gene^10^,^15^, although certain features, namely the retinal, hepatic and skeletal defects, are also found with mutations in *IFT-A* genes^16^,^17^.

The identified pathogenic mutations localize either at the beginning of the C-terminal coiled-coil domain, known to bind IFT20, another component of the IFT-B complex, or at the N-terminal domain, within the calponin homology (CH) domain, involved in tubulin binding^18^ (Fig. 1f). As most patients carried either two missense mutations, a missense mutation in association with a truncating mutation or an alternative splice mutation giving rise to partial mRNA decay, it is likely that the function of the protein is partly preserved. Indeed, the phenotypes observed in the patients bearing *TRAF3IP1* mutations somewhat reconcile the organ involvement of loss-of-function animal models, but the milder phenotypes observed suggest that the identified mutations are hypomorphic.

### *In vivo* analysis of *TRAF3IP1* mutations in zebrafish

**Figure 2:**
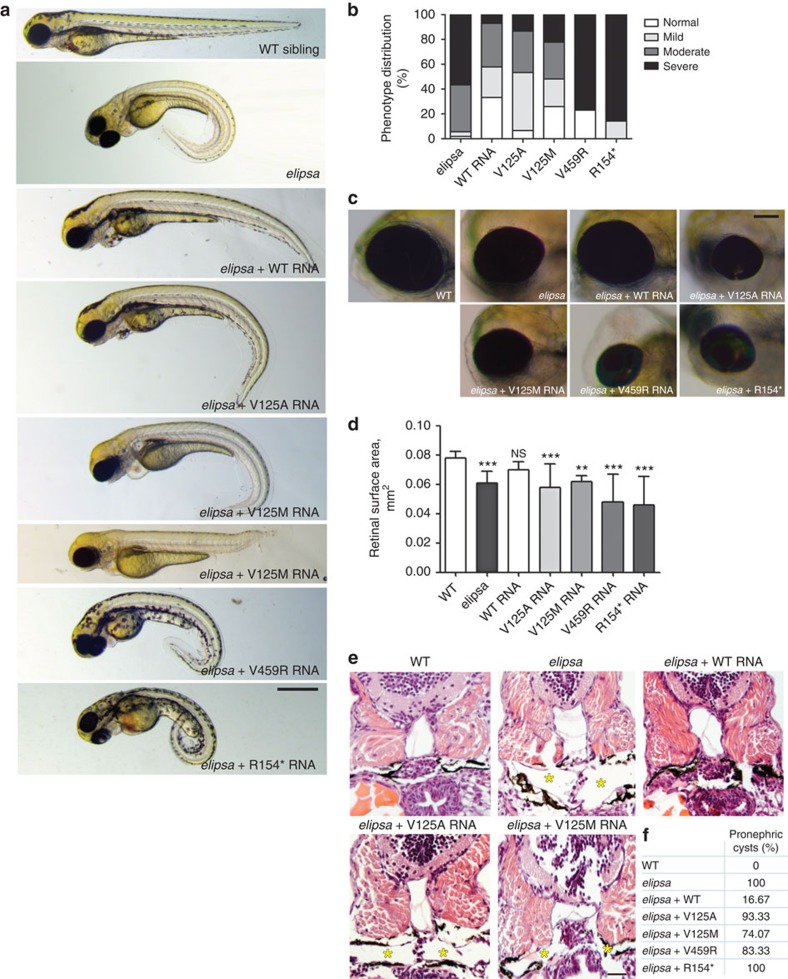
Patients mutations do not rescue the ciliopathy-associated phenotypes characteristic of *elipsa* mutants. (**a**) Lateral views of zebrafish larvae at 72 hpf of WT control, *elipsa* uninjected larvae and *elipsa* larvae injected with WT or mutant RNA constructs (p.R154* and p.V459R correspond to the human p.R155* and p.M520R mutations, respectively). Approximately 20% of V125M-injected *elipsa* larvae displayed an alternative stunted phenotype with pronephric cysts and eye defects, but lacking the characteristic body axis curvature. Scale bar, 0.5 mm. (**b**) Phenotype distribution as determined by quantification of angle of body axis curvature (*n*≥30, 4 independent experiments). (**c**) Eye phenotypes (5 dpf) of WT control, *elipsa* uninjected larvae and *elipsa* larvae injected with WT/mutant RNA constructs, lateral views, anterior to the left. Scale bar, 0.1 mm. (**d**) Surface area of the retina (mean ± s.d. of *n*=10, 2 independent experiments, *P=0.05, **P<0.01, and ***P<0.001, Dunnett’s multiple-comparison test). (**e**) H&E staining of histological cross sections of *elipsa* mutant larvae injected with WT or mutant RNA constructs at 72 hpf. Gross cystic dilations of the glomerular region extending to the pronephric tubule are indicated by asterisks. Scale bar, 20 μm. (**f**) Percentage of pronephric cysts in *elipsa* mutant larvae as well as rescued larvae (*n*≥30, 4 independent experiments).

To confirm the pathogenicity of the identified *TRAF3IP1* mutations, we injected wild type (WT) and mutated mRNA into both *elipsa* mutant and *traf3ip1* morphant zebrafish embryos. While injection of WT mRNA resulted in a partial rescue of the mutant phenotypes, injection of mutated mRNA constructs mimicking the human mutations could not rescue the curved body axis, glomerular cysts, dilated pronephric tubules, oval eye shape and loss of photoreceptors and even led to an exacerbation of the ocular phenotype (Fig. 2a–f, Supplementary Fig. 4a–f). Notably, the most consistently severe phenotypes observed were those arising from injections with the construct mimicking the p.R155* (p.R154* in zebrafish), as could be expected for a truncating mutation (Fig. 2a–f, Supplementary Fig. 4a–f). These data further prove that mutations in *TRAF3IP1* are causal for NPH and retinal degeneration.

### *TRAF3IP1* mutations result in ciliary localization defects

Having validated the damaging effects of the mutations *in vivo*, we next analysed their consequences on the localization of IFT54 within the cilia and on ciliogenesis. This study was conducted in several *in vitro* models, including patients’ fibroblasts and *Traf3ip1* knock-down (KD) mIMCD3 cells re-expressing either WT or mutant forms tagged with green fluorescent protein (GFP; Supplementary Fig. 5a,b).

**Figure 3:**
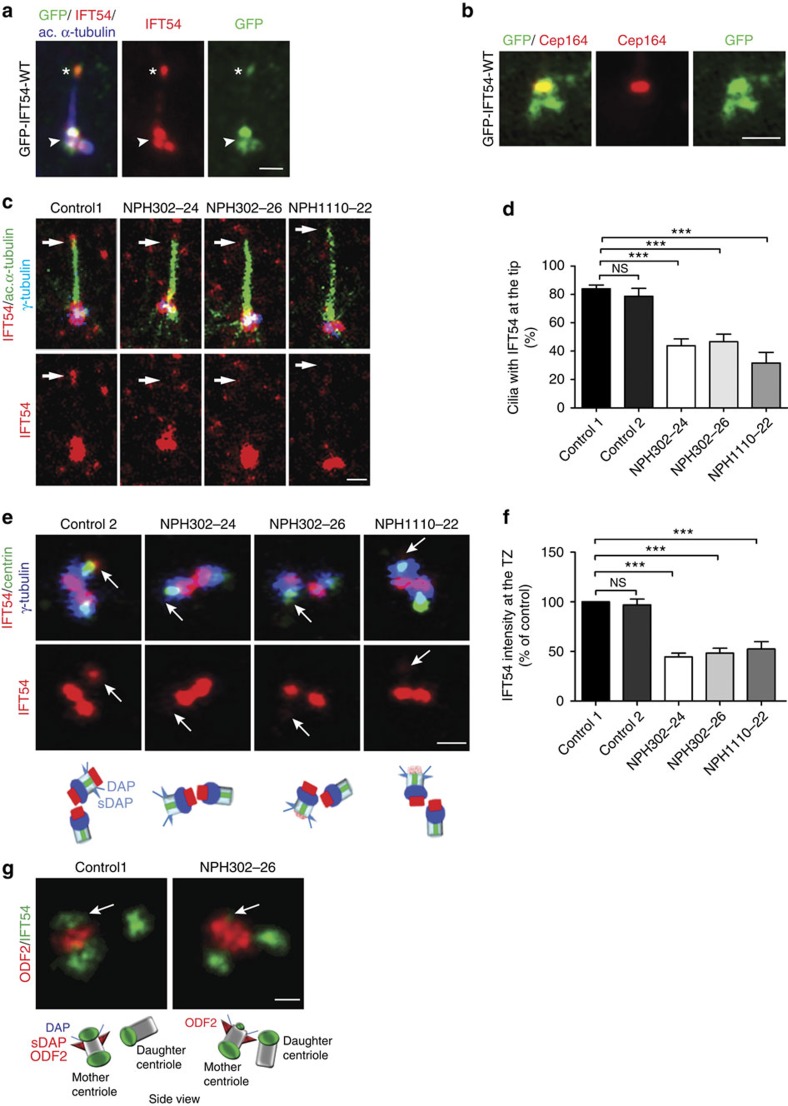
Mutations of *TRAF3IP1* impair IFT54 ciliary trafficking. (**a**) Fibroblasts transfected with GFP–IFT54-WT were fixed with MeOH and stained for GFP (green), IFT54 (red) and acetylated α-tubulin (blue, cilia). The base and the tip of the cilia are indicated by arrowhead and asterisk respectively. Scale bar, 1 μm. (**b**) *Traf3ip1*-KD mIMCD3 cells stably expressing GFP-tagged IFT54 WT were fixed with 4% PFA and stained for Cep164 (distal appendages, red). Scale bar, 1 μm. (**c**) Ciliary distribution of IFT54 in serum-starved control and patients' fibroblasts stained for IFT54 (red), acetylated α-tubulin (green, cilia) and the basal body marker γ-tubulin (blue). Scale bar, 1 μm. (**d**) Percentage of cilia with IFT54 at the distal tip of cilia (arrows in (**c**), mean ± s.e.m. of *n*=4 experiments (that is, ≈100 cilia), ***P<0.001, Bonferronni's multiple-comparison test). (**e**) Distribution of IFT54 at the basal body in ciliated fibroblasts stained for IFT54 (red) and for γ-tubulin (blue) and centrin (green), markers of proximal and distal parts of centrioles, respectively. A schematic representation of the orientation of the two centrioles, with the localization of the distal (DAP) and subdistal (sDAP) appendages is shown. Scale bar, 1 μm. (**f**) Intensity of IFT54 staining at the transition fibres/transition zone (TZ, arrows in (**e**), mean ± s.e.m. of *n*=3 experiments (that is, ≈50 cilia), ***P<0.001, Dunn's *post-hoc* test). (**g**) Fibroblasts from control or affected individuals were fixed and stained for ODF2 (red, subdistal appendages) and IFT54 (green) and analysed by STED microscopy. A schematic representation of the orientation of the analysed centrioles is shown. Arrows in **g** indicate the pool of IFT54 present at the distal tip of the mother centriole corresponding to the transition fibers/transition zone. Scale bars, 0.25 μm.

GFP–IFT54–WT was found at the base and at the tip of cilia when transfected into fibroblasts and KD mIMCD3 cells (Fig. 3a, Supplementary Fig. 5c). This localization was confirmed using a commercial anti-IFT54 antibody that specifically recognizes the human form of IFT54 (Fig. 3a, Supplementary Fig. 5b). Next, we investigated the precise localization of IFT54 at the base of cilia. It has been shown previously that IFT54 docks to the transition fibres through its interaction with FBF1 (ref. 19). As expected, IFT54 was present at the ciliary transition fibres/transition zone, based on its co-localization with the distal appendage protein Cep164 (Fig. 3b). In addition, it co-localized with γ-tubulin at the proximal part of the centrioles (Fig. 3e, Supplementary Fig. 5c). Remarkably, endogenous IFT54 in patients’ fibroblasts and IFT54 mutant forms expressed in KD mIMCD3 cells were absent from the transition zone and from the tip of cilia (Fig. 3c–g, Supplementary Fig. 5c), indicating that the mutations impair entry of IFT54 into the ciliary compartment.

Therefore, we assessed the effects of *TRAF3IP1* mutations on ciliogenesis in fibroblasts from patients. While there was no difference in the percentage of ciliated cells, cilia were significantly longer (Supplementary Fig. 6a–c). Consistently, the defects in ciliogenesis observed in *Traf3ip1*-depleted zebrafish embryos and mIMCD3-KD cells were partially rescued by re-expression of IFT54 mutants, except p.R155* (Supplementary Figs 4g and 5d). This suggests that the observed phenotype in patients as well as in zebrafish may not be caused by defects in ciliogenesis *per se*.

Moreover, in the cilia formed in patients’ fibroblasts, there was no obvious change in the localization of key ciliary proteins (IFT46, IFT140, Anks6 and Smoothened; Supplementary Fig. 6d–g), suggesting no general defect in ciliary composition and trafficking. However, we noticed a clear decrease of adenylyl cyclase III (ACIII) staining in cilia (Supplementary Fig. 7a,b). This defect in ACIII localization was associated with impaired translocation of PKA catalytic subunits from the cilium base to the cytoplasm on treatment with forskolin, an AC activator (Supplementary Fig. 7c,d). This result links IFT54 to the regulation of the cAMP/PKA pathway, as observed for mutations in *IFT172* (ref. 10). Nevertheless, the mild ciliary structural defects associated with hypomorphic mutations of *TRAF3IP1* seem insufficient to account for the large phenotypic spectrum of the patients and suggest that IFT54 may have an important extraciliary function.

### N-terminal mutations of *TRAF3IP1* impair binding to MAP4

To gain insight into the underlying mechanism responsible for the deleterious effects of *TRAF3IP1* mutations, we studied the impact of these mutations on known IFT54 interacting partners.

IFT54 forms a peripheral IFT-B sub-complex through the interaction of its C-terminal coiled-coil domain with IFT20 (ref. 20). In contrast to the two truncating alleles (p. R155* and p.M525Mfs3*), the missense mutations, including the C-terminal p.M520R, had little or no impact on IFT20 binding, indicating that the IFT54–IFT20 sub-complex is preserved in most patients (Supplementary Fig. 8a).

**Figure 4:**
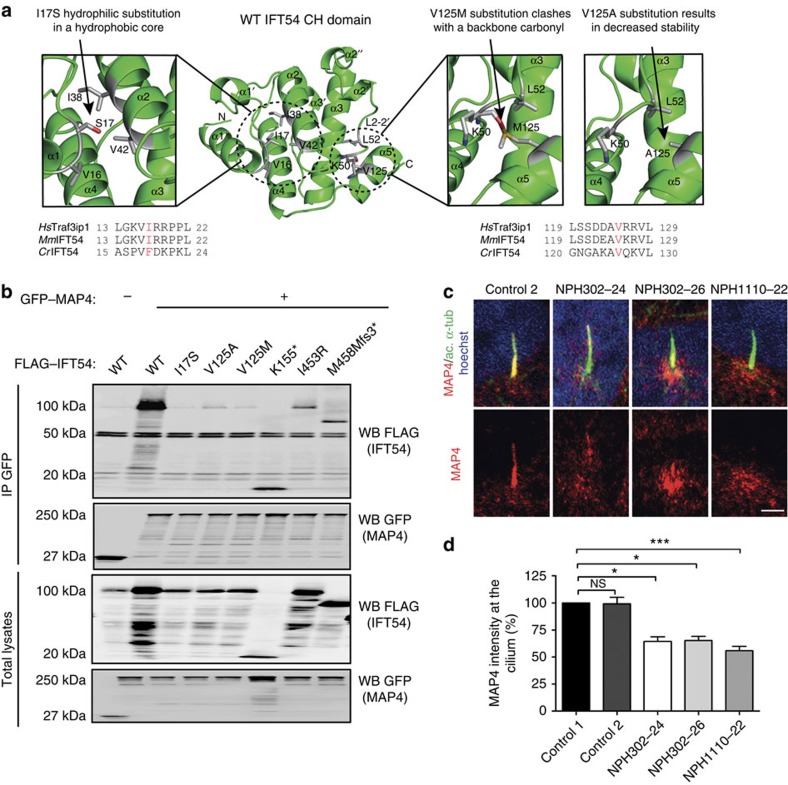
*TRAF3IP1* mutations impair folding of the CH domain and interaction with MAP4. (**a**) Crystal structure of the CH domain of *Mm*IFT54 (based on PDB entry 2EQO) showing that the I17 and V125 residues locate in conserved hydrophobic pockets (dotted line circles). The mutant residues S17, A125 and M125 were introduced (red) to show their effects on these hydrophobic pockets. (**b**) Lysates from HEK293T cells co-expressing Flag-tagged WT or mutant forms of *Mm*IFT54 (p.K155*, p.I453R and p.M458Mfs3* correspond to the human mutations p.R155*, p.M520R and p.M525Mfs3*) and GFP-MAP4 were immunoprecipitated with an anti-GFP antibody. The co-immunoprecipitation of GFP-MAP4 and Flag-IFT54 constructs was followed by western blot (WB) using GFP and Flag antibodies. (**c**) Serum-starved fibroblasts were fixed in PFA to visualize ciliary MAP4 (red; acetylated α-tubulin, green). Scale bar, 2 μm. (**d**) Intensity of ciliary MAP4 staining (mean ± s.e.m. of *n*=5 experiments (that is ≈150 cilia, *P<0.05, ***P<0.001, Dunn's *post-hoc* test).

The majority of the identified mutations are present in the N-terminal CH-domain, previously shown to be involved in the interaction with α-tubulin and potentially other cargoes for IFT transport^18^. Computational modelling of the N-terminal p.I17S and p.V125M/p.V125A substitutions predicted a disruption of two hydrophobic pockets of the CH domain (Fig. 4a). Consistently, introduction of the p.I17S or p.V125A/M mutations in this isolated CH-domain (1–133) generated insoluble (likely unfolded) recombinant proteins (Supplementary Fig. 8b–d). Moreover, circular dichroism and thermal denaturation experiments, using full-length IFT54 in complex with IFT20 (Supplementary Fig. 8e), indicated that the CH domain of the IFT54 mutants is not accurately folded at 37 °C, likely affecting IFT54 binding to α-tubulin *in vivo* (Table 2). Indeed, IFT54 can be found along cytoplasmic microtubules^21^,^22^ and we observed that structural defects incurred by N-terminal mutations effectively impaired this localization (Supplementary Fig. 9). Altogether, these data indicate that the N-terminal mutations affect the stability of the CH domain of IFT54 and therefore inhibit its ability to bind to tubulin and/or other partners.

Among several previously identified candidate partners of IFT54 (ref. 18), we found that N-terminal mutations predominantly impaired the interaction with MAP4, while WT or the p.R155* mutant display normal interactions (Fig. 4b). MAP4 is the major MAP in non-neuronal cells^23^ that stabilizes cytoplasmic microtubules^24^. MAP4 is also known to localize into primary cilia where it acts as a negative regulator of ciliary length, counteracting the function of septins^25^. Consistently, we found that ciliary MAP4 staining was drastically reduced in patients’ fibroblasts (Fig. 4c,d) suggesting that loss of interaction between MAP4 and IFT54 in N-terminal mutants results in ciliary mislocalization. This result indicates that MAP4 could be a cargo of IFT54 for ciliary import.

### *TRAF3IP1* mutations affect cytoplasmic microtubule dynamics

**Figure 5:**
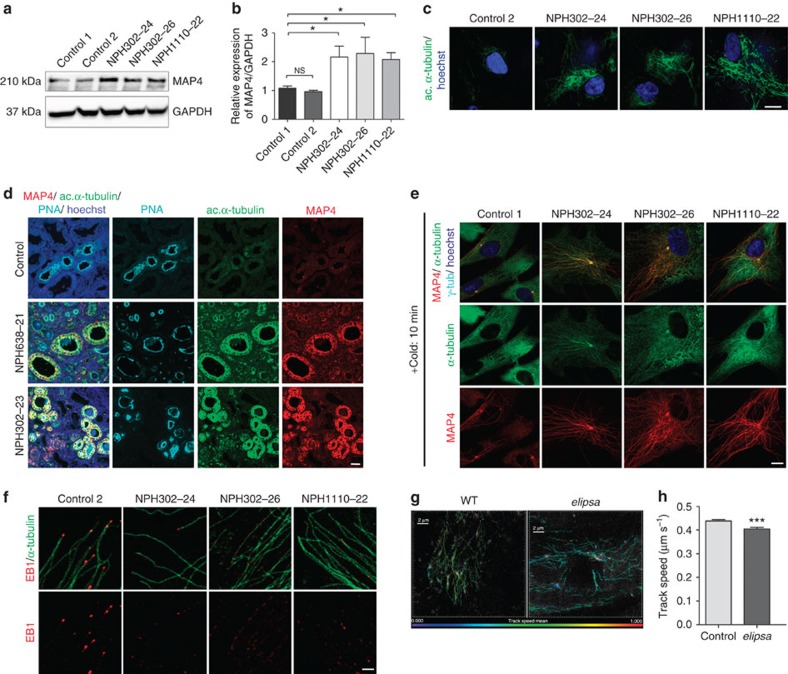
Mutations of *TRAF3IP1* increase MAP4 expression, causing cytoplasmic microtubule stabilization. (**a**) Expression of MAP4 and GAPDH in control and patients' fibroblasts were analysed by WB. (**b**) Relative expression of MAP4 normalized to that of GAPDH (mean ± s.e.m. of *n*=5 experiments, *P<0.05, Dunn's *post-hoc* test). (**c**) Fibroblasts were stained for acetylated α-tubulin (green). Scale bar, 10 μm. (**d**) Kidney biopsies from control and NPH638-21 and NPH302-23 affected individuals were stained for MAP4 (red), acetylated α-tubulin (green) and with peanut agglutinin (PNA, light blue, distal tubules). Scale bar, 25 μm. (**e**) Fibroblasts treated for 10 min on ice (to depolymerize the microtubules) were fixed with MeOH (to visualize MAP4 on microtubules) and stained for α-tubulin (green), γ-tubulin (light blue) and MAP4 (red). Scale bar, 10 μm. (**f**) Fibroblasts were stained for α-tubulin (green) and the microtubule plus-tip associated protein EB1 (red). Scale bar, 2 μm. (**g**) WT and *elipsa* embryos were injected with EB3-GFP to follow the dynamics of the growing ends of microtubules which were analysed by time lapse confocal microscopy and Imaris tracking software. Pseudo colours were used to visualize the speed of EB3 comets (from blue (slow) to red (fast)). (**h**) Track speed analysis of EB3-GFP comets in WT and *elipsa* embryos (*n*=6, mean ± s.d., ***P<0.001, *t-*test).

MAP4 is well known to bind to cytoplasmic microtubules and regulate their stability, so we analysed if *TRAF3IP1* mutations could disturb MAP4 localization and expression. Unexpectedly, MAP4 staining was strongly increased along cytoplasmic microtubules in mutant cells (Supplementary Fig. 10a), which correlates with an overall enhanced protein expression (Fig. 5a,b, Supplementary Fig. 10b–d). As MAP4 mRNA remained unchanged in mutant conditions (Supplementary Fig. 10e), it is likely that IFT54 regulates MAP4 protein stability. Hence, mutations in *TRAF3IP1* increase MAP4 expression and its recruitment to microtubules, which in turn is expected to result in their stabilization^24^.

We thus investigated whether *TRAF3IP1* mutations could affect cytoplasmic microtubule dynamics, by first assessing the level of acetylated α-tubulin, a marker of stable microtubules^26^. Hyperacetylation of α-tubulin was observed in mutant fibroblasts (Fig. 5c), as well as in *Traf3ip1*-KD mIMCD3 cells, and was restored by re-expression of IFT54-WT but not by the mutant forms (Supplementary Fig. 11a). In patients’ kidney tubules, the increase in α-tubulin acetylation correlated with enhanced MAP4 levels (Fig. 5d), indicating that *in vivo*, tubular lesions are associated with microtubule stabilization. We then evaluated the impact of *TRAF3IP1* mutations on the resistance of microtubules to cold treatment, which induces their depolymerization. Microtubules positive for MAP4 and resistant to cold treatment were detected in fibroblasts and mIMCD3 mutant cells (Fig. 5e, Supplementary Fig. 11b), consistent with increased microtubule stability. Finally, we examined the level of EB1 staining at the microtubule plus-tips, which reflects the dynamics of growing ends of microtubules^27^. EB1 staining at the plus-tips of microtubules was drastically reduced in mutant fibroblasts (Fig. 5f), mimicking the overexpression of GFP-MAP4 in control fibroblasts (Supplementary Fig. 12a). Altogether, these results demonstrate that mutant cells display an abnormal stability of cytoplasmic microtubules that is linked to the increased expression of MAP4.

To further confirm these observations, we studied the consequences of *Traf3ip1* mutation on microtubule dynamics *in vivo*, using an EB3-GFP fusion construct as a reporter of plus-tip dynamics in *elipsa* mutant and WT zebrafish. Mutant embryos displayed a significantly lower speed of EB3 comets, confirming slower rates of microtubule polymerization compared with heterozygous control siblings (Fig. 5g,h). Altogether, these data emphasize a novel role for IFT54 as a negative regulator of microtubule stability, likely through the regulation of MAP4.

### IFT54 is required for correct epithelial morphogenesis

**Figure 6:**
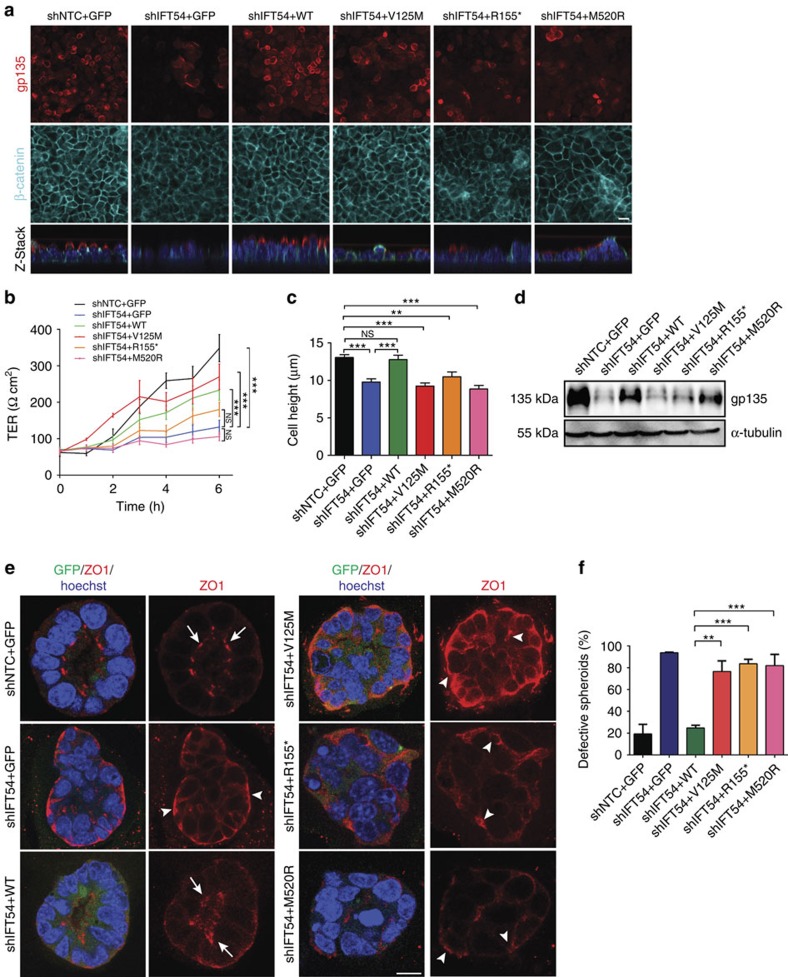
*TRAF3IP1* mutations lead to epithelialization and polarity defects. (**a**) mIMCD3 cells grown until confluence on filters were subjected to Ca^2+^-free medium to disrupt the tight junctions. Six hours after Ca^2+^ addition, cells were analysed by immunofluorescence using the apical marker Gp135 (red) and β-catenin (light blue) to stain the cell junctions. Scale bar, 10 μm. (**b**) Following Ca^2+^ switch, tight junction re-formation was assessed by measurement of trans-epithelial resistance (TER) at different time points (mean ± s.e.m. of *n*=5 independent experiments, two-way ANOVA; NS: not-significant, ***P<0.001 at 6 h). (**c**) Height of mIMCD3 cells grown on filters measured as the distance from the base to the top of the cells (GFP staining, not shown; mean ± s.d. of *n*≥20, from 3 independent experiments, ***P<0.001, Bonferonni's multiple-comparison test). (**d**) Expression of the apical marker Gp135 was analysed by Western blot with α-tubulin as a loading control. (**e**) mIMCD3 cells grown in matrigel 3D matrix to form spheroids were stained for ZO1 (tight junctions, red) and analysed by confocal microscopy. Arrows indicate ZO-1 at the apical junctions, while arrow heads point to mislocalized ZO-1. Equatorial sections of representative spheres are shown for each cell line. Scale bars, 10 μm. (**f**) Percentage of abnormal spheroids (no/small lumen filled with cells) (mean ± s.d., *n*=80 spheroids from 2 independent experiments, ***P≤0,001, **P<0.002, Bonferonni's multiple-comparison test).

Microtubule network architecture is crucial for epithelial integrity^28^,^29^, hence we investigated the impact of *TRAF3IP1* mutations on epithelialization. The microtubule network appeared disorganized in polarized *Traf3ip1*-KD mIMCD3 cells, and was restored by re-expression of IFT54-WT but not by IFT54 mutants (Supplementary Fig. 11c). The reformation of tight junctions and cell polarity were assessed by trans-epithelial resistance (TER) measurement following Ca^2+^ switch (Fig. 6a–d). *Traf3ip1*-KD cells presented a decreased TER and reduced β-catenin localization at cell junctions, both of which were partially rescued by re-expression of WT and the p.V125M mutant but not by the p.R155* or p.M520R mutants (Fig. 6a,b). In addition, *Traf3ip1*-KD cells and all cells re-expressing IFT54-mutant proteins appeared flatter than controls and displayed decreased expression of the apical marker Gp135 (Fig. 6a,c,d). These results suggest that IFT54 mutant proteins may perturb the establishment of cell junctions and/or the targeting of apical proteins during epithelialization, as we previously reported for NPHP1 and NPHP4 (ref. 30).

To further study epithelialization and polarity processes, we used a well-established three-dimensional (3D) spheroid culture system that reflects the biology of kidney tubular epithelial cells. When cultured in matrigel, mIMCD3 control cells formed single-lumen spheroids, whereas *Traf3ip1*-KD cells formed abnormal structures with small lumens filled with dividing cells and/or surrounding misarranged nuclei, with markedly altered expression of the tight-junction component ZO1 (Fig. 6e,f). Normal lumen formation and ZO1 localization were restored by re-expression of IFT54-WT but not by any of the IFT54 mutant proteins (Fig. 6e,f). These results demonstrate that in addition to its known function in ciliogenesis, IFT54 plays a key role in the early steps of epithelial morphogenesis, a process independent of cilia^31^.

### Decreased MAP4 expression rescues TRAF3IP1 mutation defects

Our data suggest that the epithelialization defects associated with *TRAF3IP1* mutations are mediated by the observed attendant increase in cytoplasmic MAP4 expression and microtubule stability. To validate this hypothesis, we first studied the consequences of overexpression of MAP4. A stable cell line overexpressing MAP4 displayed microtubule hyperacetylation and defects in cell-polarity establishment, associated with a loss of the apical marker Gp135 and of β-catenin at the cell junctions (Supplementary Fig. 12b–d). Finally, MAP4 overexpressing cells formed abnormal 3D structures with no lumens (Supplementary Fig. 12e). Thus, increased levels of MAP4 expression recapitulates the epithelialization and polarity defects observed in *Traf3ip1* mutant cells.

**Figure 7:**
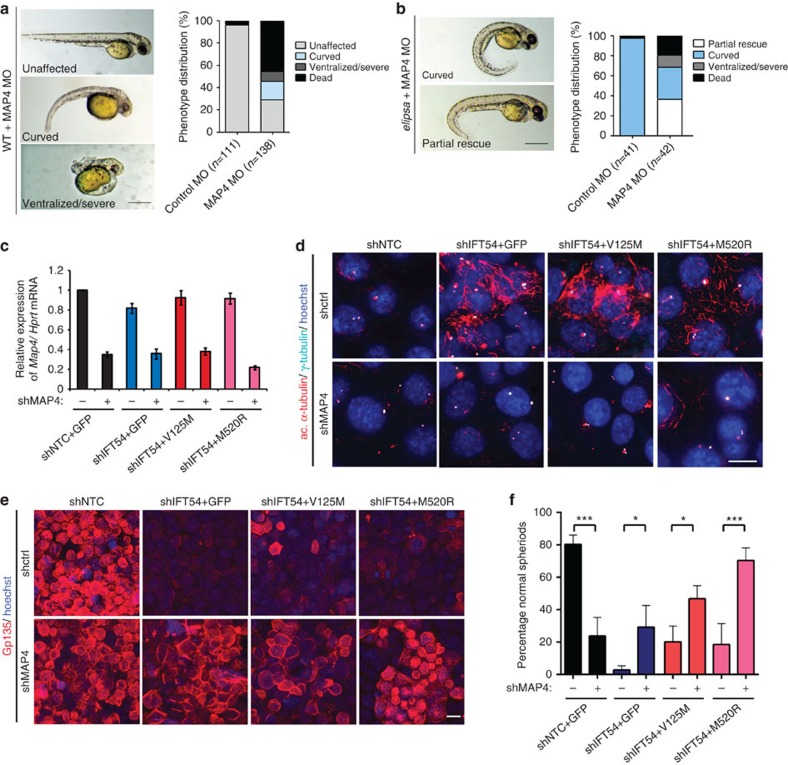
Defects in *TRAF3IP1* mutants are mediated by MAP4. (**a**) Lateral views of WT zebrafish embryos injected with *map4* morpholino at 48 hpf and phenotype distribution in WT embryos injected with control or *map4* morpholino. (**b**) Lateral views of *elipsa* zebrafish embryos injected with *map4* morpholino at 48 hpf and phenotype distribution in *elipsa* mutant embryos injected with control or *map4* morpholino (data shown as combined result of *n*=3 independent experiments). Scale bars, 1 mm. (**c**) Relative expression of *Map4* normalized to that of *Hprt* was analysed by qPCR in control and *Traf3ip1*-KD mIMCD3 cells stably expressing GFP or GFP-IFT54 mutants and *Map4* shRNA. (**d**) Control and *Traf3ip1*-KD/ *Map4*-KD mIMCD3 cells expressing either GFP or IFT54-GFP fusions were fixed in MeOH and stained for acetylated α-tubulin (red) and γ-tubulin (light blue). Scale bar, 10 μm. (**e**) Six hours after Ca^2+^ switch, mIMCD3 cells grown until confluence on filters were fixed with 4% PFA and stained for the apical marker Gp135 (red). Scale bar, 10 μm. (**f**) Percentage of normal spheroids of control and *Traf3ip1*-KD/ *Map4*-KD mIMCD3 cells expressing either GFP or IFT54-GFP fusions grown on Matrigel for 5 days (mean ± s.d., *n*≥100 spheroids from 3 independent experiments, ***P≤0,0001, *P<0.012, Bonferonni's multiple-comparison test).

To provide further evidence that *TRAF3IP1-*related defects were mediated by MAP4, *map4* was knocked down in zebrafish using morpholinos. Loss of MAP4 results in very high mortality rates, severe ventralization or curved body axis phenotypes in the vast majority of WT embryos (>70%) (Fig. 7a). In contrast, a much smaller proportion (>20%) of *elipsa* embryos are fatally affected by loss of MAP4. More strikingly, 40% of *map4*-KD *elipsa* embryos showed a reduction in the severity of the curved body axis phenotype at 48 hpf and almost a third showed no increase in severity of phenotype (Fig. 7b). A 5-base-pair mismatch control morpholino had no effect on >90% of injected *elipsa* embryos or WT siblings (Fig. 7a,b). Therefore, our results indicate that the *elipsa* phenotype is partially induced through dysregulation of MAP4 expression.

We next inactivated *Map4* by shRNA in mIMCD3 *Traf3ip1*-KD cells (Fig. 7c). The decreased expression of MAP4 in *Traf3ip1*-KD cells and in cells re-expressing the mutants reduced microtubule acetylation to a level similar to that of control cells (Fig. 7d). In addition, knocking down *Map4* rescued Gp135 expression in both *Traf3ip1*-KD cells and in cells re-expressing the mutants (Fig. 7e). Finally, *Map4*-KD partially restored the formation of normal spheroids in *Traf3ip1*-KD cells, and in cells re-expressing the mutant forms of IFT54 (Fig. 7f). Overall these results show that normalization of MAP4 overexpression can rescue to a large extent the cellular phenotype observed in *Traf3ip1*-KD cells. It is noteworthy that depletion of MAP4 in control cells caused abnormal sphere formation similar to that seen when overexpressing MAP4 and mirroring our observations in WT zebrafish. This suggests that precise regulation of MAP4 expression level is essential for correct epithelialization of kidney collecting duct cells.

Altogether, these results demonstrate that *TRAF3IP1* mutations lead to abnormal microtubule dynamics and altered cell polarity, via defective regulation of MAP4.

## Discussion

In this work, we have identified mutations of *TRAF3IP1* as a cause of NPH and retinal degeneration, associated with liver fibrosis, skeletal abnormalities and obesity. We thus propose *NPHP20* as an alias for *TRAF3IP1.* Of the sixteen IFT-B components, IFT54 is only the fourth component demonstrated to be causative of human ciliopathies. Previous loss-of-function studies have also shown the importance of this anterograde IFT subunit, as loss of *Traf3ip1* is embryonic lethal with attendant neural, skeletal and ocular defects in mice, and loss of cilia, retinal degeneration, body axis curvature and pronephric cysts in *elipsa* mutant zebrafish^11^,^12^,^13^.

Although IFT54 has been shown to be required for ciliogenesis, the patients’ mutations identified in this study do not impair cilia formation. This is indicative of a hypomorphic effect of these mutations, that may explain the milder phenotype observed in patients compared with loss-of-function animal models. Analysis of ciliary composition from mutant fibroblasts did not reveal major defects, except for ACIII, which was strongly decreased and associated with a defective cAMP/PKA pathway. Defective cAMP/PKA signalling is a common feature of mutations in *IFT* genes^10^,^32^ and provides a potential explanation for the obesity observed in patients, a characteristic of an ACIII-deficient murine model^33^. It could also explain the increased cilia length observed in patient fibroblasts, as the ACIII–cAMP pathway has been linked to cilia length regulation^34^.

Because of this relatively mild phenotype, we investigated additional potential cargoes of IFT54 that could be affected by the identified hypomorphic mutations. After screening a large number of candidates from literature, we demonstrated that N-terminal mutations of *TRAF3IP1* abrogate the interaction with MAP4 and suppress its ciliary entry, suggesting MAP4 as a cargo for IFT54. Since we have previously described MAP4 as a negative regulator of ciliogenesis^25^, the decreased ciliary expression of MAP4 may also be linked to the increased cilia length observed in patients' fibroblasts.

Most surprisingly, although the level of ciliary MAP4 protein is decreased, we observed a strong increase in MAP4 staining along cytoplasmic microtubules coupled with an overall increase in protein expression. Thus it seems that IFT54 negatively regulates MAP4 protein levels and may inhibit recruitment of MAP4 to the microtubules, a process known to induce microtubule stabilization^24^. Indeed, in both our *in vivo* and *in vitro* models, we observed that mutations in *TRAF3IP1* result in increased stabilization of microtubules. These defects in microtubule dynamics were associated with loss of apico-basal polarity in epithelialized kidney cells. Importantly, overexpression of MAP4 reproduces similar cellular defects while knock-down of *Map4* rescues *TRAF3IP1* mutations for most of the cellular phenotypes observed, demonstrating that IFT54 plays a role outside the cilia, by modulating MAP4 levels and thereby regulating cytoplasmic microtubule dynamics and cell polarity establishment/maintenance (Supplementary Fig. 13).

Although it is likely that IFT54 modulates MAP4 degradation, the exact mechanism by which IFT54 regulates MAP4 remains to be determined. As demonstrated for Septins^23^, it is possible that IFT54 competes with MAP4 for binding to the microtubules. Indeed, the association between the CH domain of IFT54 and MAP4 could be indirect through microtubules. Thus, N-terminal mutants of IFT54, which may have lower affinity for tubulin (as indicated by their impaired localization to microtubules and their unfolded CH domain), would increase the number of available binding sites for MAP4 on microtubules. As a consequence, MAP4 may be stabilized when bound to microtubules, promoting its accumulation. On the other hand, the p.M520R mutant still associates with microtubules and with MAP4. However, introduction of this mutation leads to increased MAP4 levels, suggesting the C-terminal domain of IFT54 may recruit an additional player required for MAP4 degradation. MAP4 has been shown to be phosphorylated by several microtubule affinity-regulating kinases^24^ as well as by PKA, resulting in MAP4 dissociation from the microtubules^35^. Thus, the low PKA activity in mutant cells could also provide an explanation for increased MAP4 levels along the microtubules.

By competing with Septin2, MAP4 has been shown to impair the delivery of proteins from the Golgi to the plasma membrane, a process required for the establishment of polarized/columnar shape epithelia^36^. In a similar manner, IFT54 could play a role in the transport of membrane and junction proteins along the microtubule network, with *TRAF3IP1* mutations resulting in mistargeting of Gp135 and β-catenin. The subsequent defect in polarity establishment may lead to loss of differentiation and progressive degeneration of the epithelium, thus explaining the tubulo-interstitial lesions observed in NPH, the retinal degeneration and the development of hepatic fibrosis, common progressive features of the so-called ‘ciliopathies’.

In the course of this study, we observed that decreased MAP4 expression is deleterious for lumen formation in 3D-cultured control cells and is fatal to WT zebrafish embryos, whereas it appears to have a partial rescue effect on *Traf3ip1*-KD cells and *elipsa* mutants. In humans, MAP4 loss-of-function mutations have recently been associated with Seckel syndrome (microcephaly at birth, dwarfism, brachydactyly or cone-shaped epiphyses)^37^. Conversely, we showed that overexpression of MAP4 results in similar epithelialization phenotypes to those observed in *TRAF3IP1* mutant cells indicating that *TRAF3IP1-*dependent increase of MAP4 expression results in NPH, retinal degeneration and hepatic fibrosis. Therefore, fine regulation of MAP4 appears to be essential for proper tissue homeostasis.

Taken together, our data highlight two not mutually exclusive functions of IFT54: first, as a regulator of ciliary composition; and second, as a negative regulator of cytoplasmic microtubule stability via MAP4. Since other NPHPs have been reported to localize along the cytoplasmic microtubule network, and hyperacetylation remains an unexplained observation in several ciliopathy models^11^,^38^,^39^,^40^,^41^,^42^, defective regulation of microtubule dynamics may be a general pathophysiological mechanism leading to diverse degenerative organ lesions, in particular NPH. Further investigation is needed to establish whether NPHPs share a common aetiology linked to MAP4 and ciliary cAMP signalling as described in our study. Such understanding could provide a new perspective for potential therapeutics for ciliopathies.

## Methods

### Patients and families

Written informed consent was obtained for all individuals enrolled in this study and approved by the Institutional Review boards at the University of Paris Descartes and at the University of Michigan.

### Homozygosity mapping, exome sequencing and mutation calling

Homozygosity mapping in families NPH302 and A4336 was performed using ‘Human Mapping 250k NspI’ array and parametric logarithm of odds scores were calculated with MERLIN software^43^ for NPH302, and GENEHUNTER 2.1 (ref. 44) ALLEGRO^45^ for A4336, assuming autosomal-recessive inheritance.

WES in patients A4336-22 and NPH302-23 were performed as follow. In brief, genomic DNA was isolated from blood lymphocytes and subjected to exome capture using Agilent SureSelect human exome capture arrays (Life Technologies) followed by next generation sequencing on the Illumina sequencing platform. Ciliary exome-targeted sequencing was conducted in NPH579-22, NPH638-21 and NPH1110-22, using a custom SureSelect capture kit (Agilent Technologies) targeting 4.5 Mb of 20,168 exons (1 221 ciliary candidate genes), including *TRAF3IP1* (refs 14, 46). Briefly, Agilent SureSelect librairies were prepared from 3 μg of 300 genomic DNA samples sheared with a Covaris S2 Ultrasonicator according to manufacturer’s instructions. The SOLiD molecular barcodes for traceable ID of samples were added at the end of the capture step. The Ovation Ultralow System (NuGEN Technologies) was used to prepare HiSeq2500 precapture barcoded libraries. The ciliome capture by hybridization was performed on a pool of 10 to 16 barcoded precapture libraries. Sequencing performed on SOLiD5500XL (Life Technologies) and HiSeq2500 (Illumina) was done on pools of barcoded ciliome libraries (64 barcoded ciliome libraries per SOLiD FlowChip and 16 ciliome libraries per lane of HiSeq FlowCell). Paired-end reads were generated (75+35 for SOLiD, 100+100 for HiSeq) and sequences were aligned to the reference human genome hg19 with Illumina’s processing software ELAND (CASAVA 1.8.2), the Burrows–Wheeler Aligner (Illumina) or mapread (SoliD).

Downstream processing for WES in NPH302-23 and ciliome in NPH579-22, NPH638-21 and NPH1110-22 was carried out with the Genome Analysis Toolkit (GATK), SAMtools, and Picard Tools, following documented best practices ( http://www.broadinstitute.org/gatk/guide/topic?name=best-practices). All variants were annotated using a software system developed by the Paris Descartes University Bioinformatics platform^47^. The mean depth of coverage obtained was greater than × 90, and >89% of the exome was covered at least × 15. Different filters were applied to exclude all variants located in non-exonic regions, pseudogenes, untranslated regions or known polymorphic variants with a frequency above 1%, that is present in databases such as dbSNP, 1000 genome projects and all variants identified by in-house exome sequencing (5,150 exomes and 1,020 ciliomes). The functional consequence of missense variants was predicted using SIFT ( http://sift.jcvi.org/www/SIFT_enst_submit.html), CADD/PHRED2 ( http://cadd.gs.washington.edu/) and PolyPhen2 software ( http://genetics.bwh.harvard.edu/pph2/). For A4336-22, SAMtools37 was used to call single-nucleotide variants and insertion/deletion at targeted bases. Variants with minor allele frequencies <1% in the Yale (1,972 European subjects), NHLBI GO Exome Sequencing Project (4,300 European and 2,202 African American subjects), dbSNP (version 135) or 1,000 Genomes (1,094 subjects of various ethnicities) databases were selected and annotated for impact on the encoded protein and for conservation of the reference base and amino acid among orthologs across phylogeny. Sequence reads were mapped to the human reference genome assembly (GRCh37/hg19) using CLC Genomics Workbench (version 4.7.2) software (CLC bio, Aarhus, Denmark). Mutation calling was performed by geneticists/cell biologists, who had knowledge of the clinical phenotypes and pedigree structure, as well as experience with homozygosity mapping and exome evaluation, as in Boyden *et al.*^48^

Sanger sequencing using the primers described in Supplementary Table 3 was performed to validate the NGS findings and the segregation of the mutation within all the families. To confirm segregation of the two heterozygous mutations (both located on exon 4) carried by patient NPH638-2 and for which parental DNA was not available, exon 4 was amplified by PCR and subcloned into a pGEM-T Easy vector. Sequencing of the independent clones revealed that the mutations were never found in the same PCR fragment, thus demonstrating that each of the mutations were on a different allele.

### Purification and pull down of recombinant proteins

Truncations of *Mm*IFT54 containing the N-terminal CH domain (either WT or point mutations) were cloned into bacterial pEC vectors with cleavable glutathione *S*-transferase or hexahistidine tags using ligation-independent cloning. These pEC vectors were created at the Department for Structural Cell Biology at the Max Planck Institute of Biochemistry (details available on request)^49^ and expressed in the *Escherichia coli* BL21 (DE3) Gold pLysS strain. *E.coli* cells were lysed by sonication in 50 mM Tris-HCL pH 7.5, 150 mM NaCl, 5 mM β-mercaptoethanol and 10% glycerol. The lysates were incubated with BSA-blocked Ni^2+^–NTA or GSH beads for 1 h followed by three washes with lysis buffer. Bound material was eluted in lysis buffer supplemented with 500 mM imidazole (Ni^2+^–NTA beads) or 30 mM reduced glutathione (GSH-beads). Large-scale purification of the *Mm*IFT54 CH-domain included the additional steps (after affinity-tag cleavage using tobacco etch virus protease) of anion-exchange chromatography (MonoQ, GE healthcare) and size exclusion chromatography (HiLoad75, GE healthcare) in 10 mM HEPES pH 7.5, 150 mM NaCl and 1 mM DTT. All samples were analysed using SDS–PAGE.

For heterodimeric IFT54/20 complex purification, full-length *Cr*IFT54 (WT or point mutants) and *Cr*IFT20 were cloned either untagged or with tobacco etch virus-cleavable HIS-tag into pFL vectors. After producing viral particles by transfection of Sf21 insect cells using CellFectin (Life Technologies), IFT20/54 (WT or V126A/M) were expressed by infection of HighFive insect cells (Life Technologies)^49^. The cells were homogenized using a Dounce Homogenizer in a 20 mM HEPES pH 7.5 buffer containing 250 mM sucrose, 5 mM β-mercaptoethanol, 10 mM KCl, 1.5 mM MgCl_2_ and protease inhibitor cocktail (Roche) and then purified in large scale as described above.

### Circular dichroism spectroscopy

Secondary structure content was analysed on a Jasco J-715 spectropolarimeter at 4 °C using 0.1 mg ml^−1^ of recombinant purified proteins in a 0.1-cm quartz cuvette. The measurements were performed in 10 mM HEPES 7.5, 100 mM NaCl, 10% glycerol and 5 mM DTT. Data were obtained and processed using the Spectra Manager v2.06 software from Jasco. The measured curves were buffer corrected and secondary structure assignments were done using the CONTIN fitting method and SMP56 as the reference protein set. Melting curves were measured continuously from 10 to 90 °C, with additional full spectra taken in 10 °C steps. Data analysis was performed in Spectra Manager v2.06.

### Zebrafish strains and morpholinos

Adult zebrafish were maintained at 28 °C, in system water with a conductivity of 500 μS and a pH of 7. Embryos were cultured at 28 °C in embryo medium with 0.1% w/v methylene blue. The *elipsa tp49d* mutant, which encodes a premature stop codon at position 195 (previously described^13^) was obtained as a gift from J. Malicki. Heterozygous sibling embryos were used as controls for all experiments using the *elipsa* mutant line. An anti-sense morpholino targeting *traf3ip1* (previously published^13^) was used for knock-down experiments. Knock-down of *map4* was performed using an ATG translation-blocking morpholino (5′-GGCATCACGTAAACTAAAGTCCATC-3′) and a 5-base-pair mismatch (5′-GACATAACGTAAAATAAAATCAATC-3′) control, designed by and ordered from Genetools, LLC. Wild-type Tü:AB fish were used for all morpholino experiments unless stated otherwise.

To rescue the *elipsa/traf3ip1* knock-down phenotype, full-length zebrafish *ift54* coding sequence was amplified by reverese transcription PCR (RT–PCR). Site-directed mutagenesis was then used to introduce mutations at the desired locations. The resulting products were then cloned into the pGEM-T Easy vector and constructs were linearized and transcribed using the SP6/T7 mMessage mMachine kit (Ambion). Approximately 100 pg of mRNA was injected into embryos at the 1-cell stage.

Effects of RNA injections (WT and mutated RNAs) were evaluated based on severity of body curvature, analysis of pronephric cilia, presence or absence of pronephric cysts and surface area of the retina. Body curvature was quantified by measuring the internal angle of each larva using ImageJ software. Larvae were classified as follows: severe (0–60°); moderate (60–90°); mild (90–120°); and normal (over 120°). Live embryos and larvae were photographed using a Leica M165FC microscope and camera. For histological analysis, larvae were fixed in 4% paraformaldehyde (PFA, Electron Microscopy Sciences), embedded in paraffin and sectioned at 5 μm. Sections were stained with haematoxylin and eosin (H&E) and photographed with a Nikon DXM1200F camera and an Olympus BX41 microscope.

### Plasmids and establishment of stable cell lines

*Mm*Flag-*Traf3ip1* construct was a gift from G. Pazour^20^. Human cDNA of *TRAF3IP1* (Invitrogen) was cloned into the pcDNA–DEST40 vector. The mutations were created using the QuickChange site-directed mutagenesis kit according to the manufacturers protocol (Stratagene). For gene silencing of *TRAF3IP1*, the shRNA sequences described in Supplementary Table 3 were cloned into the lentiviral pLKO.1 vector that contained a cassette conferring puromycin resistance. mIMCD3 (from ATCC) were transduced with non-targeted (shNTC) or *Traf3ip1*-specific shRNA sequences and selected by adding puromycin (Sigma, 2 μg ml^−1^) to the culture medium (DMEM/F12(1:1) with GlutaMaxI medium containing 10% fetal bovine serum (FBS), 100 U ml^−1^ penicillin and 100 mg ml^−1^ streptomycin (all from Life technologies). For rescue experiments, shNTC and sh*Traf3ip1*(shRNA #461) mIMCD3 cells were transfected with pDEST40-GFP-*TRAF3IP1*-WT or mutant plasmids using Amaxa Cell Line Nucleofector (Solution V, program O17, Lonza)^50^, sorted by FACS and selected with 0.35 mg/ml G418 (Life Technologies). shNTC and *Traf3ip1*-KD cells expressing either GFP or GFP-IFT54 mutants were re-transduced with control or *Map4* shRNA sequences (MSH027680-LVRH1GH, GeneCopeia) and selected with 200 μg/ml hygromycin B (Life Tehnologies). Fibroblasts were obtained from skin biopsies of patients and cultured in Optimem supplemented with 10% FBS, 100 U ml^−1^ penicillin and 100 mg ml^−1^ streptomycin (all from Life Technologies). Fibroblasts were then cultured up to 10 passages. Ciliogenesis was induced by starving the cells in serum-free Optimem for 24 h. HEK293T cells (from ATCC) were cultured in DMEM supplemented with 10% FBS, 100 U ml^−1^ penicillin and 100 mg ml^−1^ streptomycin.

### Antibodies

The following antibodies were used in the study: acetylated α-tubulin (6–11-B-1, used at 1:10,000 to detect cilia and 1:1,000 to detect cytoplasmic microtubules) and α-tubulin (T5168, used at 1:1,000 for immunofluorescence (IF) and 1:50,000 for western blot) from Sigma; EB1 (610534, used at 1:200), β-catenin (610153, used at 1:200) and anti-PKAc (610980, used at 1:200) from BD biosciences; acetylated α-tubulin (ab24610, used at 1:500) and α-tubulin (ab18251, used at 1:500) from Abcam; IFT54 (HPA037858, Atlas Antibodies, used at 1:50 for IF and 1:1,000 for WB); ZO1 (61–7300, used at 1:100) from Life Technologies; ARL13B (17711-1-AP, Proteintech, used at 1:400); Gp135 (AF1556, R&D, used at 1:200 for IF and 1:1000 for WB); γ-tubulin (DQ-19, Sigma, used at 1:500); γ-tubulin (C-20, used at 1:200), MAP4 (H-300 and G-10, used at 1:400 for IF and 1:1,000 for WB) and ACIII (C-20, used at 1:200) from Santa Cruz; GAPDH (MAB374, used at 1:4000 for WB) from Millipore. Highly cross adsorbed secondary antibodies (Alexa Fluor 488, Alexa Fluor 546, AlexaFluor 555, AlexaFluor 532 and Alexa Fluor 647) were obtained from Molecular Probes (Life Technologies) and were used at 1:200 dilution.

### Immunofluorescence and image analysis

Zebrafish embryos at 48 hpf were fixed overnight at 4 °C in 4% PFA, washed in PBS (Sigma) and incubated in PBS-Triton-4% BSA (Sigma) for 1 h at 4 °C before antibody incubation. Alternatively, fibroblasts and mIMCD3 cells were fixed in 4% PFA, permeabilized with 0.2% Triton-X 100 or fixed in ice-cold Methanol for 5 min and incubated with 1% skim milk or 1% BSA, 0.1% Tween20 before incubation with primary (1–3 h at room temperature or overnight at 4 °C) and secondary (30 min at room temperature) antibodies. Appropriate controls were performed omitting the primary antibodies. DNA was stained with Dapi or Hoechst (Life Technologies) (except for STED imaging). Confocal images were taken on either Zeiss LSM 700 or LEICA SP8 microscopes. Images were analysed with ImageJ. Alternatively, super resolution images were acquired using a LEICA SP8 gSTED microcoscope, equipped with a 660-nm laser that quenches the fluorescence outside the centre of the focus. Images were then deconvoluted using Huygens software. Ciliogenesis analyses were performed on a CV7000 confocal microscope from YOKOGAWA with × 40 long distance. Z-stacks were acquired with identical acquisition settings (gain, offset, laser power) and all measurements of fluorescence intensity were performed on maximum intensity projection, calculated with the Yokogawa software. For all cilia numerical values (frequency, length), we developed one specific pipeline using CellProfiler software^51^. In brief, nuclei were detected as primary objects using Otsu Adaptive two-class thresholding. For cilia length, a mask was constructed by applying MoG global thresholding on ARL13B staining, followed by measurement of the major axis length. Cilia frequency was calculated by dividing cilia and nuclei counts. All data points are performed in duplicate with four fields acquired per well, and an average of 300 cells per field. Cell profiler pipeline was run using the Linux cluster interface JENKINS. Spheroids were directly analysed using the ZEN 2011 software (Zeiss).

### Calcium switch assay and TER measurement

mIMCD3 cells grown on 65-mm Transwell filters (Corning) for 7 days were subjected to Ca^2+^ switch as described in Straight *et al.*^52^ Briefly, cells were washed with PBS-4 mM EGTA and Ca^2+^ free DMEM (Life Technologies) containing 4 mM EGTA was added to the cells for 45 min to disrupt cell junctions. Cells were then washed twice with normal culture medium (DMEM/F12) and TER was determined using a Millicell-ERS volt-ohm meter (Millipore) immediately after the addition of normal growth medium and at the indicated time points. Six hours after Ca^2+^ switch, cells were fixed with 4% PFA and processed as described above.

### Spheroid assay

Three-dimensional spheroid cell culture was performed using 5% Matrigel (BD) in chamber slides (Lab-TEK). Ten thousand cells were plated per chamber and spheroids were grown at 37 °C/5% CO_2_ for 5 days. Spheroids were fixed with 1% PFA, further stained and analysed as described in ‘immunofluorescence analysis’.

### Protein extraction and western blotting

Cells were extracted in 50 mM Tris-HCl, 150 mM NaCl, 0.5% sodium deoxycholate, 2 mM EDTA, 1% Triton X-100, 0.1% sodium dodecyl sulfate and protease inhibitor cocktail (Roche). Protein quantification was then performed using the BCA protein assay kit (ThermoScientific). A measure of 30–50 μg of proteins were loaded on 8 or 10% acrylamide gels, blotted on PVDF membrane (Millipore) and the membrane was incubated using the indicated antibodies. Western blots were then analysed with Bioprofil software.

### qPCR

Total cellular mRNA was isolated using Qiagen Extraction Kit and treated with DNase I. 1 μg of total RNA was reverse-transcribed using Superscript II (Life Technologies). Relative expression levels of the *TRAF3IP1* or *MAP4* mRNAs were determined by real-time PCR using either Absolute SYBR Green ROX Mix (ABgene) or TaqMan Gene Expression Assay (Applied Biosystems) with specific primers (Supplementary Table 3). *HhTRAF3IP1, HhMAP4, MmTraf3ip1* (Mm01285632_m1, Life Technologies) expression performed in triplicate was normalized to *HhGAPDH* or *MmTbp* (Mm00446971_m1, Life Technologies) mRNA expression. Data were analysed with the 2^−ΔΔCt^ method^30^.

### Co-immunoprecipitation

HEK 293T cells were co-transfected with Flag-tagged *MmTraf3ip1* constructs and GFP–*MmIft20* (kindly given by G. Pazour) or GFP-*MmMap4* (gift from J. Nelson) using the calcium phosphate method. Forty-eight hours post transfection, cells were lysed in 50 mM Tris-HCl pH 7.5, 150 mM NaCl, 0.5% Triton and lysates were first incubated with rabbit or mouse isotypic control antibodies and G-protein beads (Sigma) for 1 h at 4 °C. Precleared lysates (containing 1 mg of proteins) were then incubated with rabbit anti-Flag (Sigma) or mouse monoclonal anti-GFP antibodies (Roche) coupled to G-protein beads for 3 h at 4 °C. Beads were then washed three times with increasing amounts of NaCl (150 nM; 300 nM and 600 nM NaCl in 50 mM Tris-HCl pH 7.5), resuspended in 2 × sample buffer (Sigma) and boiled for 5 min. Western blot analyses were conducted as described above.

### Microtubule tracking

EB3–GFP plasmid (obtained from R. Köster) was linearized and reverse transcribed using the SP6 mMessage mMachine kit (Ambion). *Elipsa* embryos were then injected with 50 pg of EB3-GFP RNA at the 1-cell stage, and photographed at 60 hpf. Time-lapse confocal microscopy images were recorded over a period of 5 min, and the resulting sequences were analysed using Imaris software to quantify the microtubule dynamics *in vivo*.

### Statistical analyses

Results are presented as means of at least *n*=2 independent experiments±s.e./s.d. Statistical analyses were performed with the GraphPad Prism software by using ANOVA followed by Bonferonni’s or Dunnett’s multiple-comparisons test versus a control group (*post hoc*) or by using Kruskal–Wallis test followed by Dunn’s multiple comparisons *post hoc* test. *P*<0.05 was considered statistically significant.

## Additional information

**How to cite this article:** Bizet, A. A. *et al.* Mutations in TRAF3IP1/IFT54 reveal a new role for IFT proteins in microtubule stabilization. *Nat. Commun.* 6:8666 doi: 10.1038/ncomms9666 (2015).

## Supplementary Material

Supplementary Information Supplementary Figures 1-14 and Supplementary Tables 1-3

## Figures and Tables

**Table 1:**
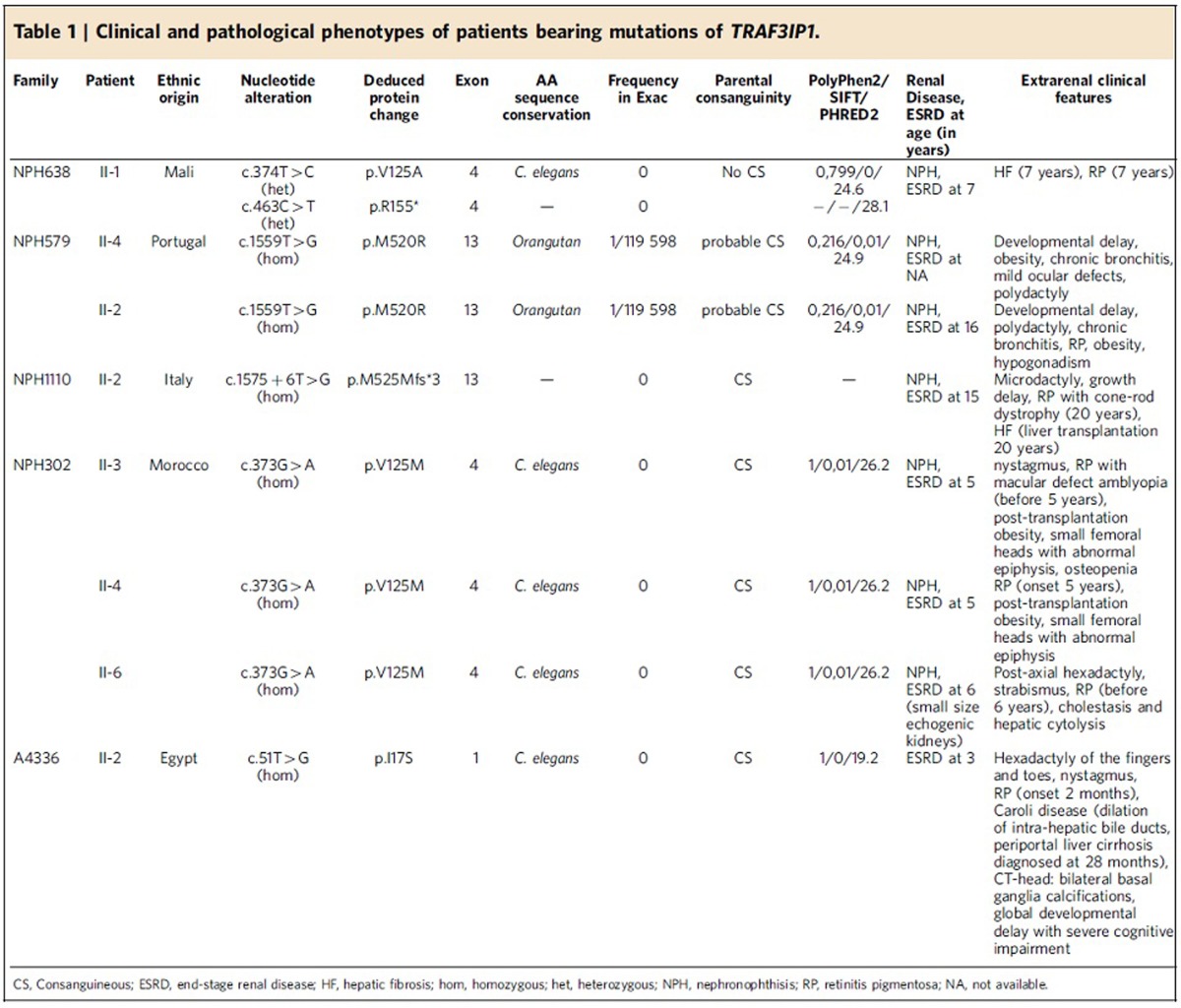
Clinical and pathological phenotypes of patients bearing mutations of *TRAF3IP1*.

**Table 2:** *TRAF3IP1* N-terminal mutations result in lower stability of the IFT54/IFT20 complex.

**Sample**	**Secondary structure determination**				**Thermal unfolding**
	**α (%)**	**β (%)**	**Turn (%)**	**Unstructured (%)**	**Melting temperature TM (°C,** ± **s.d.)**
*Cr*IFT54-WT/*Cr*IFT20	46	9	16	29	51.15±0.040
*Cr*IFT54-V126A/*Cr*IFT20	39	11	19	31	48.75±0.061
*Cr*IFT54-V126M/*Cr*IFT20	35	16	18	31	48.15±0.106
*Cr*IFT20	71	1	6	22	41.62±0.146

Secondary structure determination and thermal unfolding using circular dichroism spectroscopy for WT as well as p.V126A and p.V126M mutants of CrIFT54 (corresponding to the human p.V125A and p.V125M mutations) in complex with *Cr*IFT20.
