# Supplementary material for: Mutations in TRAF3IP1/IFT54 reveal a new role for IFT proteins in microtubule stabilization
Source: Nat Commun. 2015 Oct 21;6:8666. doi: 10.1038/ncomms9666 (PMC4617596; doi:10.1038/ncomms9666)
Supplement: Supplementary Information — Supplementary Figures 1-14 and Supplementary Tables 1-3 [file ncomms9666-s1.pdf]

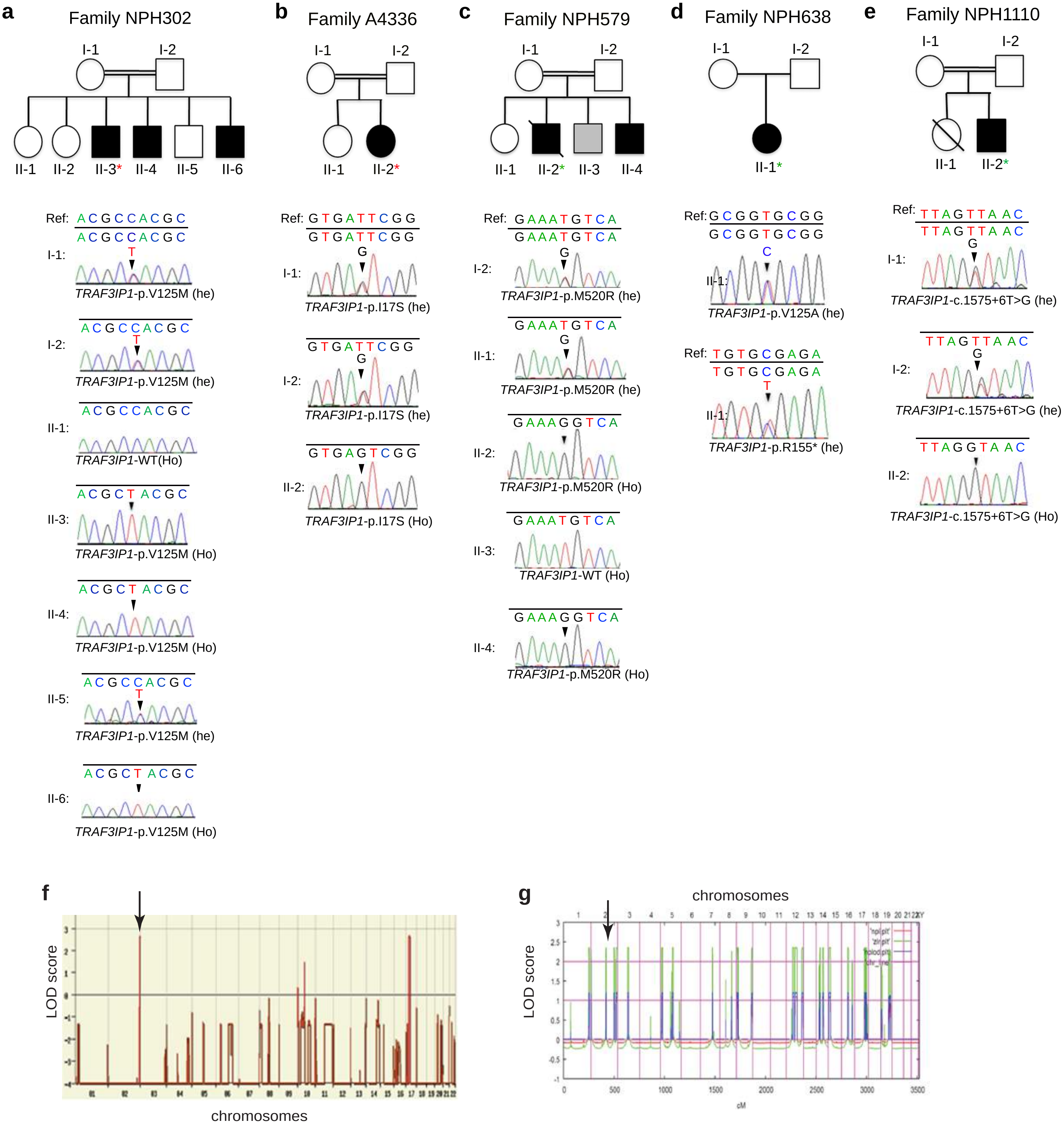

Supplementary Figure 1: Sequence and linkage analysis. (**a-e**) Pedigree of families with mutations in *TRAF3IP1*. The individual II-3 (grey box) in family NPH579 presents a phenotype unrelated to *TRAF3IP1* mutations: microcephaly and polymalformation syndrome. WES or "ciliome"sequencing was performed on individuals indicated by a red or a green asterisk, respectively. Below each pedigree, the sequence trace with identified mutated nucleotide is shown. (**f-g**) Linkage analysis of families NPH302 (**f**) and A4336 (**g**) revealed homozygous regions of 15MB and 211MB respectively. A maximum logarithm of odds (LOD) scores of 2.6557 (**f**) and 2.4 (**g**) was obtained at the *TRAF3IP1* locus (arrow).

a

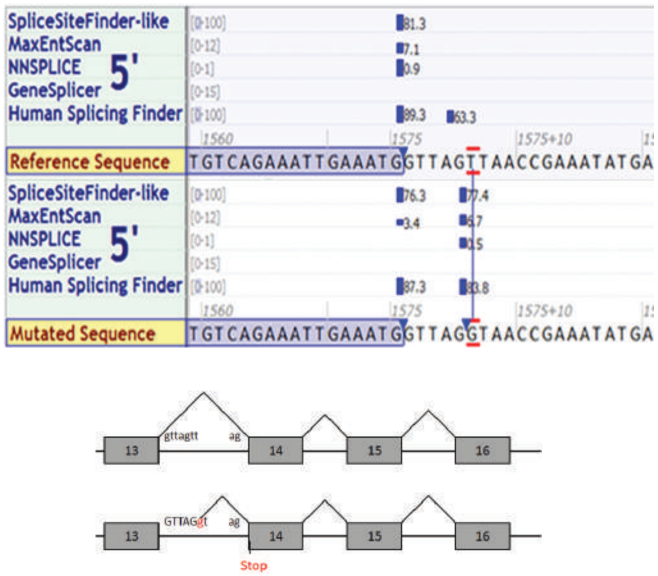

b

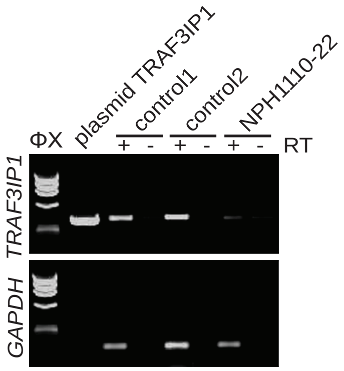

c

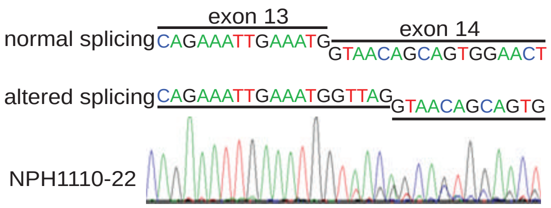

d

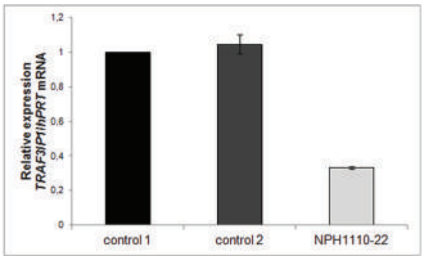

e

```
Homo Sapiens              1 M----NAAVVRRTQEALGKVIRRPPLTEKLLSKPPFRYLHDIITEV 42
Pongo Abelii              1 M----NAAVVRRTQEALGKVIRRPPLTEKLLSKPPFRYLHDIITEV 42
Mus musculus              1 M----NAAVVRRTQEALGKVIRRPPLTEKLLNKPPFRYLHDIITEV 42
Danio rerio               1 M----NESVAKKTQETLGKVIKKPPLTEKLLSKPPFRYLHDIFSEV 42
Drosophilia melanogaster  1 MSEKDLDAIILETQKVLGKYIKKPPLTEKLLKKPPFRFLMDVFSNF 46
Caenorhabditis elegans    1 MS-------VEETREILEKVIQKPQLTDQLLSRPPFKFIVDIVSNV 39
Chlamydomonas reinhardtii 1 M--CDNWQATIDTLQGASPVFDKPKLSQKLLEKPPFRFLHDVVTAV 44
```

f

```
Homo Sapiens              106 ELLQIIGKCCLNKLSSDDAVRRVLAGEKGEVKGRASLTSRSQ 148
Pongo Abelii              106 ELLQIIGKCCLNKLSSDDAVRRVLAGEKGEVKGRASLTSRSQ 148
Mus musculus              106 ELLQLIGKCCLSKLSSDEAVKRVLAGDKGDSRGRAQRTSKAQ 148
Danio rerio               106 ELLQVIAKCCLNKLSSDEAVKRVLAGDKLDQKGKPS-TSRSQ 147
Drosophilia melanogaster  110 ELLQAMASVAEKNLEWDSIVDQVV------------------ 134
Caenorhabditis elegans    101 KMLQMLGT---NATSFN------------------SRNGTGE 122
Chlamydomonas reinhardtii 108 IFLQMLGRAC-QKGNGAKAVQKVLGG---------------- 133
```

g

```
Homo Sapiens              508 QFVV---EAAPQLSEMSEIEMVTAVELEEEEKHGGLVKKILE 548
Pongo Abelii              508 QFVV---EAAPQLSEMSEIEMVTAVEL-EEEKHGGLVKKILE 547
Mus musculus              441 QFVV---EAAPQLSEIADIDMVPSGELEDEEKHGGLVKKILE 482
Danio rerio               446 QFVVe--EAAPPPSDVPEVES-NSLELQGDDKHGGLVKKILE 487
Drosophilia melanogaster  420 NLVIIEDSAAHDIVEGGGDEPALEGQLDAQ---GRLVQQILE 461
Caenorhabditis elegans    364 NFIMe--NDEEDGDRTARIED-----LVDEEDRGALVQKIME 401
Chlamydomonas reinhardtii 326 EVVH---EQTPVLSGGANMT----------GEQGVLVKDILA 357
```

h

Homo sapiens	*Danio rerio*	*Mus musculus*	*Chlamydomonas reinhardtii*
p.I17S (H)		p.I17S	
p.V125A (h)	p.V125A	p.V125A	p.V126A
p.V125M (H)	p.V125M	p.V125M	p.V126M
p.R155* (h)	p.R154*	p.K155*	
p.M520R (H)	p.V459R	p.I453R	
p.M525Mfs*3 (H)		p.M458Mfs3*	
	elipsa tp49d mutant ; p.195*		

Supplementary Figure 2: Pathogenicity of the c.1575+6T>G mutation identified in individual NPHP1110-22 and amino acid conservation of the mutated residues.(**a**) In individual NPH1110-22, the identified c.1575+6T>G mutation on intron 13 creates a new donor splice site (recognized by 4 different softwares on Alamut) with higher scores than the “normal” one (0.5 vs 0 on NNsplice, 6.7 vs 3.4 on MaxEntScan). This leads to a premature STOP codon (p.M525Mfs*3). (**b**) RT-PCR analysis of the *TRAF3IP1* transcript in patient NPH1110-22 fibroblasts and control individuals using primers located in exons 9 and 16. A single band of 509 bp was detected from controls and individual NPH1110-22 cDNAs. (**c**) Sanger sequencing of the NPH1110-22 RT-PCR product revealed that the c.1575+6T>G mutation leads to partial aberrant splicing: the normal splicing sequence is detected as well as the altered splicing product. (**d**) q-PCR demonstrating decreased expression of *TRAF3IP1* mRNA in individual NPH1110-22 compared to controls. Altogether, these results indicate that the aberrant spliced RNA from individual NPH1110-22 undergoes RNA decay. (**e-g**) Sequence alignment showing conservation of the mutated residues p.I17 (**e**), p. V125 (**f**) and p.M520 (**g**). (**h**). Table recapitulating all the modeled mutations used.

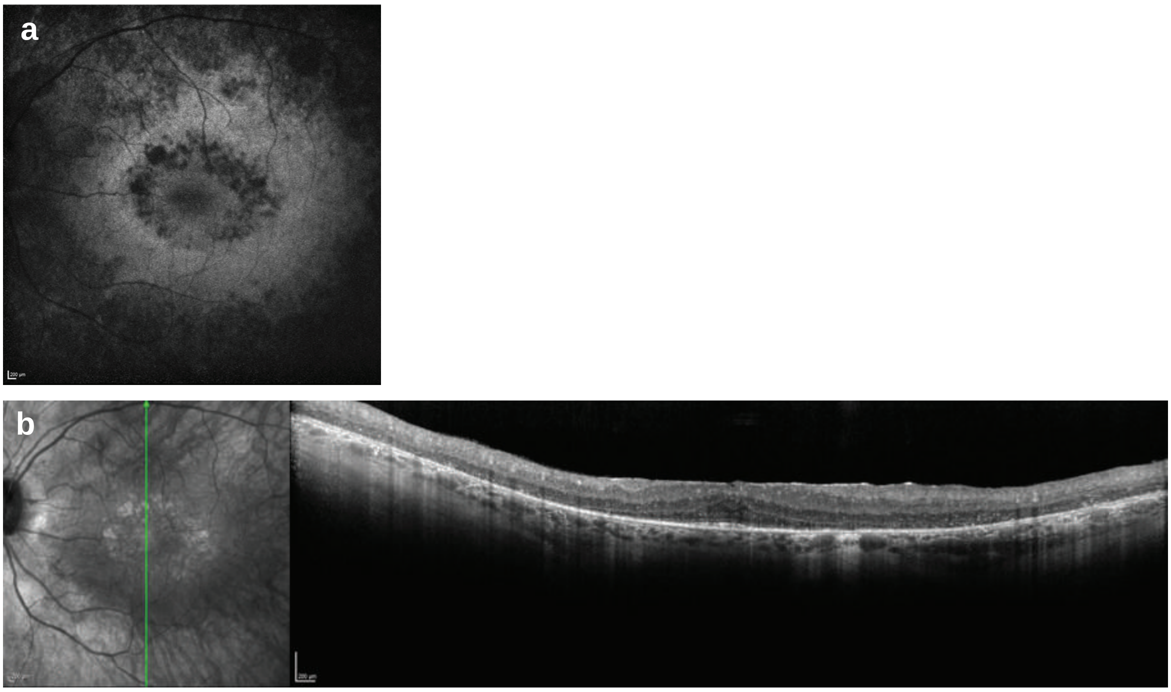

Supplementary Figure 3: Retinal defects in affected individual NPHP1110-22. (**a**) Autofluorescence of the fundus of individual NPH1110-22 showing diffuse peripheric hypo-autofluorescences and a relatively unaffected posterior pole of the retina, yet with a ring of perimacular hypo-autofluorescences surrounded by sprinkled hyper-autofluorescence. (**b**) Optical coherence tomography (OCT, NPH1110-22) showing retinal reduction marked by loss of the deep layers corresponding to photoreceptors. The fovea is erased due to traction exerted by the retinal membrane.

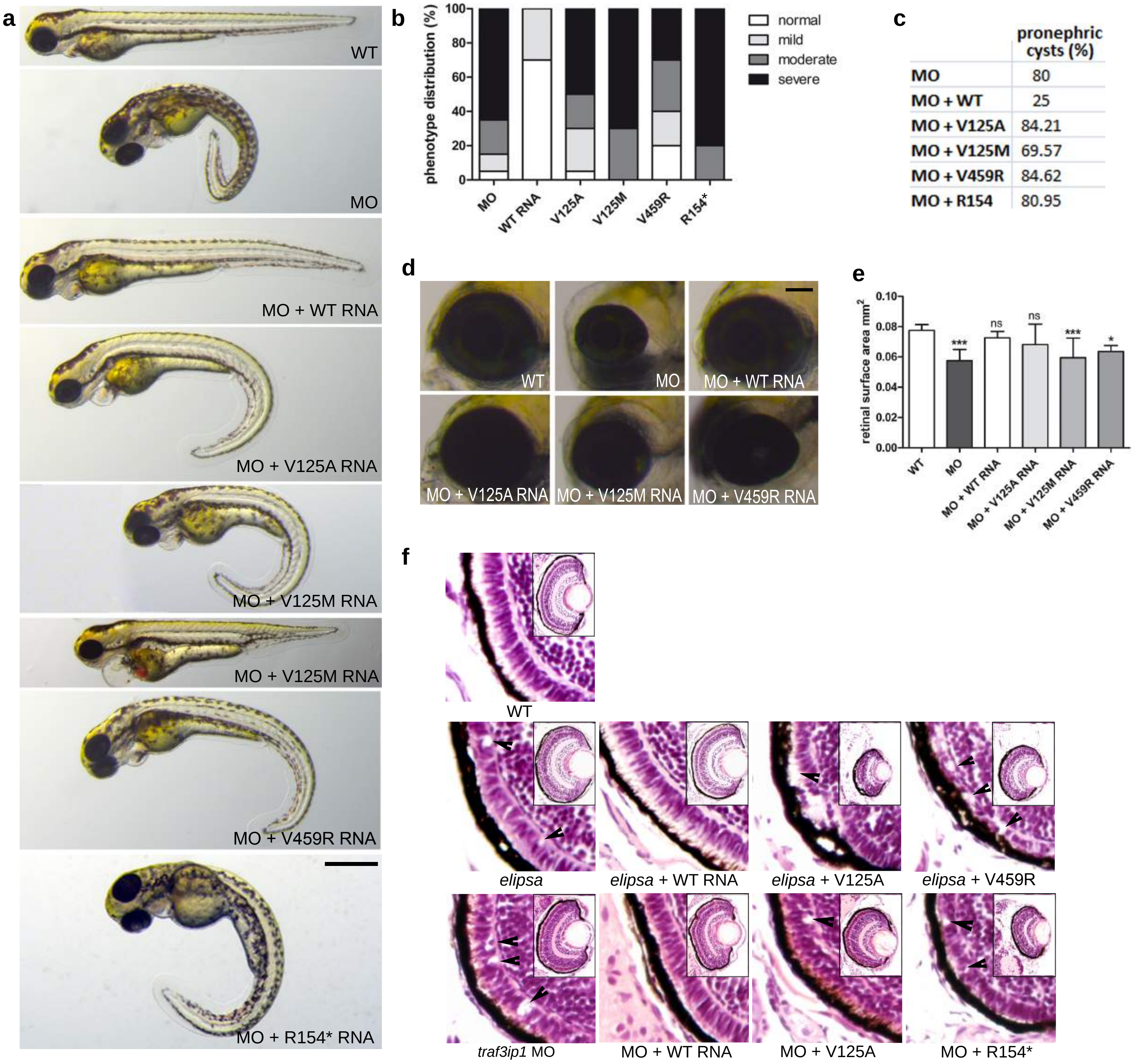

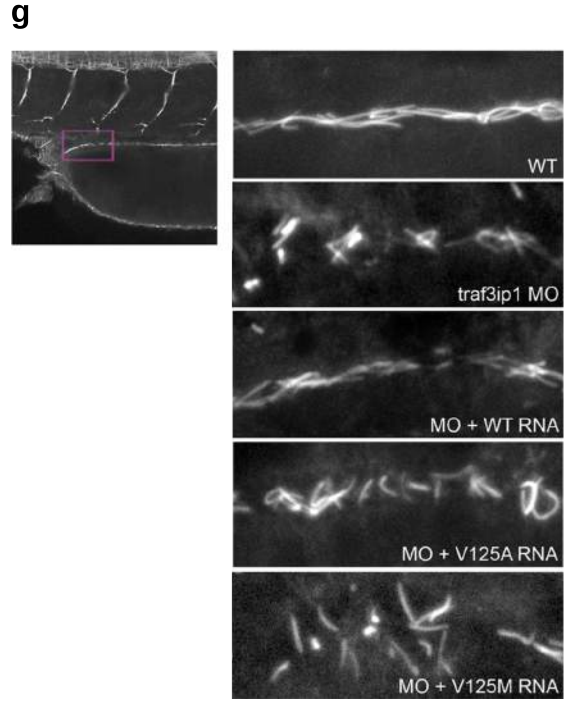

Supplementary Figure 4: Patient mutations do not rescue the kidney and retinal phenotypes characteristic of *traf3ip1* morphant larvae. (**a**) Body axis curvature phenotype of *traf3ip1* morphant larvae (72 hpf) and morphant larvae co-injected with WT and mutant *traf3ip1* RNA. Scale bar, 0.5 mm. (**b**) Phenotype distribution as determined by quantification of angle of body axis curvature (n=20, 3 independent experiments). (**c**) Percentage of pronephric cysts in morphant and rescued embryos (n≥20, 3 independent experiments). (**d**) Eye phenotypes (5 dpf) of WT, *traf3ip1* morphant larvae, and larvae co-injected with *traf3ip1* morpholino and WT/mutant RNA constructs. Scale bar, 0.1 mm. (**e**) Surface area of the retina (mean +/- SD of n=10 from 2 independent experiments, *P<0.05, **P< 0.01 and ***P< 0.001, Dunnett's multiple-comparison test) (**f**) H&E staining of histological cross sections through the eyes of elipsa mutant larvae and *traf3ip1* morphant larvae injected with WT or mutant RNAs. Loss of photoreceptor cells is indicated by arrowheads. (**g**) Cilia in the pronephric duct (48 hpf) of indicated morphants were stained for acetylated-α-tubulin (n=9, 3 independent experiments).

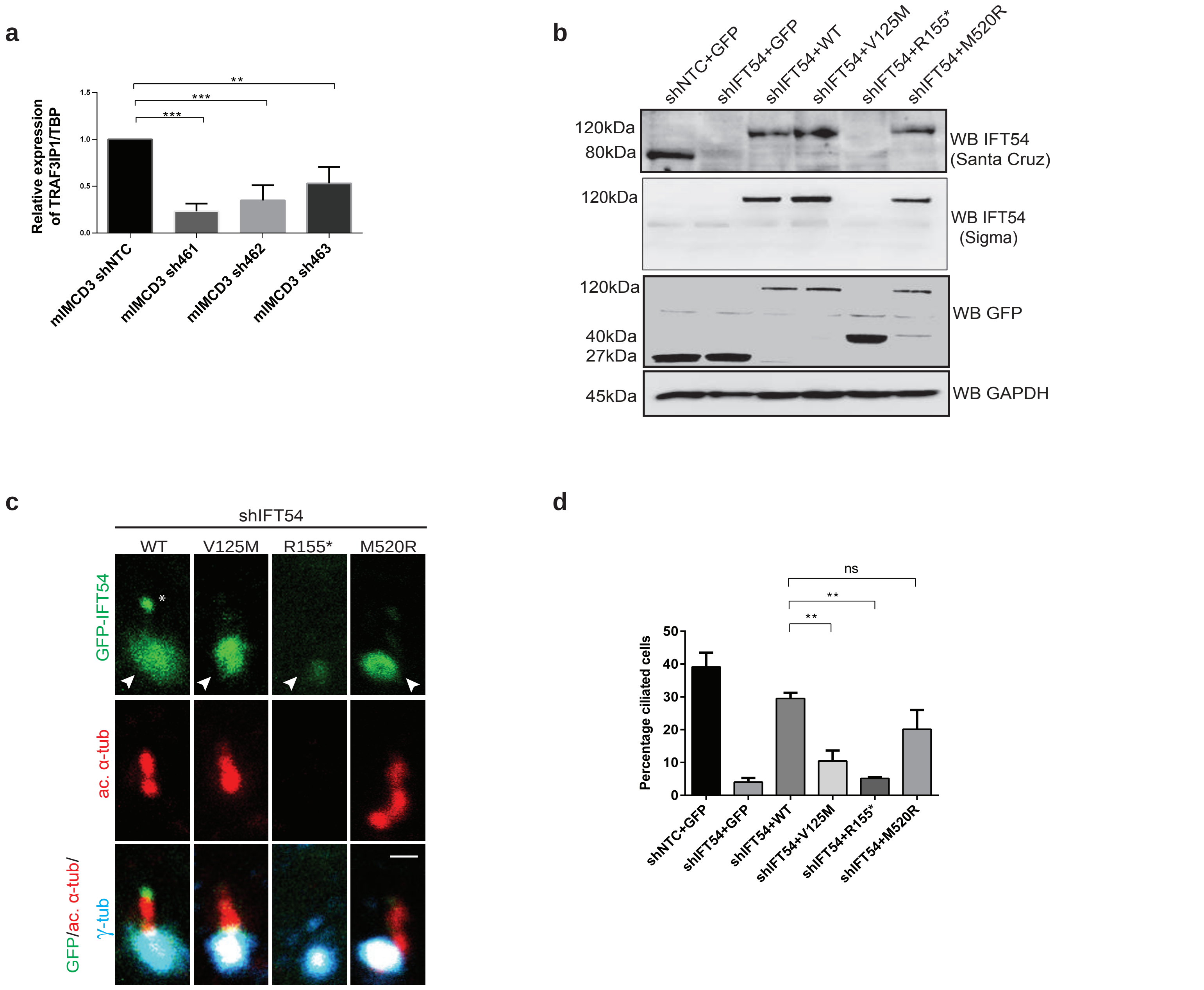

Supplementary Figure 5: Ciliary localization and rescue in *Traf3ip1* KD cells of IFT54-WT and mutants. (**a**) Relative expression of *Traf3ip1* in shRNA knockdown mIMCD3 clones was analysed by qRT-PCR and normalized to *Tbp* (mean +/- SD of n=3 experiments, **P<0.005, ***P<0.0007, Bonferonni's-multiple-comparison-test). (**b**) Expression of IFT54 (top), GFP-fusions (middle) and GAPDH (bottom) in *Traf3ip1* knockdown mIMCD3 cells (shIFT54) stably expressing GFP-tagged IFT54 WT or mutant proteins (green) was analyzed by Western blot (WB) using an IFT54 antibody from Santa Cruz, which recognizes both the human and murine form of IFT54 and using an IFT54 antibody from Sigma which is specific for the human (transfected form) of IFT54.
(**c**) *Traf3ip1*-KD mIMCD3 cells (shIFT54) stably expressing GFP-tagged IFT54 WT or indicated mutant proteins were fixed and stained for acetylated α-tubulin (cilium, red) and γ-tubulin (basal body, light blue). GFP stainings at the basal body region and the tip of cilia are indicated by arrowheads and asterisks, respectively. Scale bar, 1 μm. (**d**) Quantification of ciliated cells in *Traf3ip1* KD mIMCD3 stably expressing GFP-tagged IFT54 WT or mutant proteins stained for Arl13b (n cells > 500, mean +/- SD of n=2 experiments, ns = not-significant, **P<0.006, Bonferonni's-multiple-comparison-test).

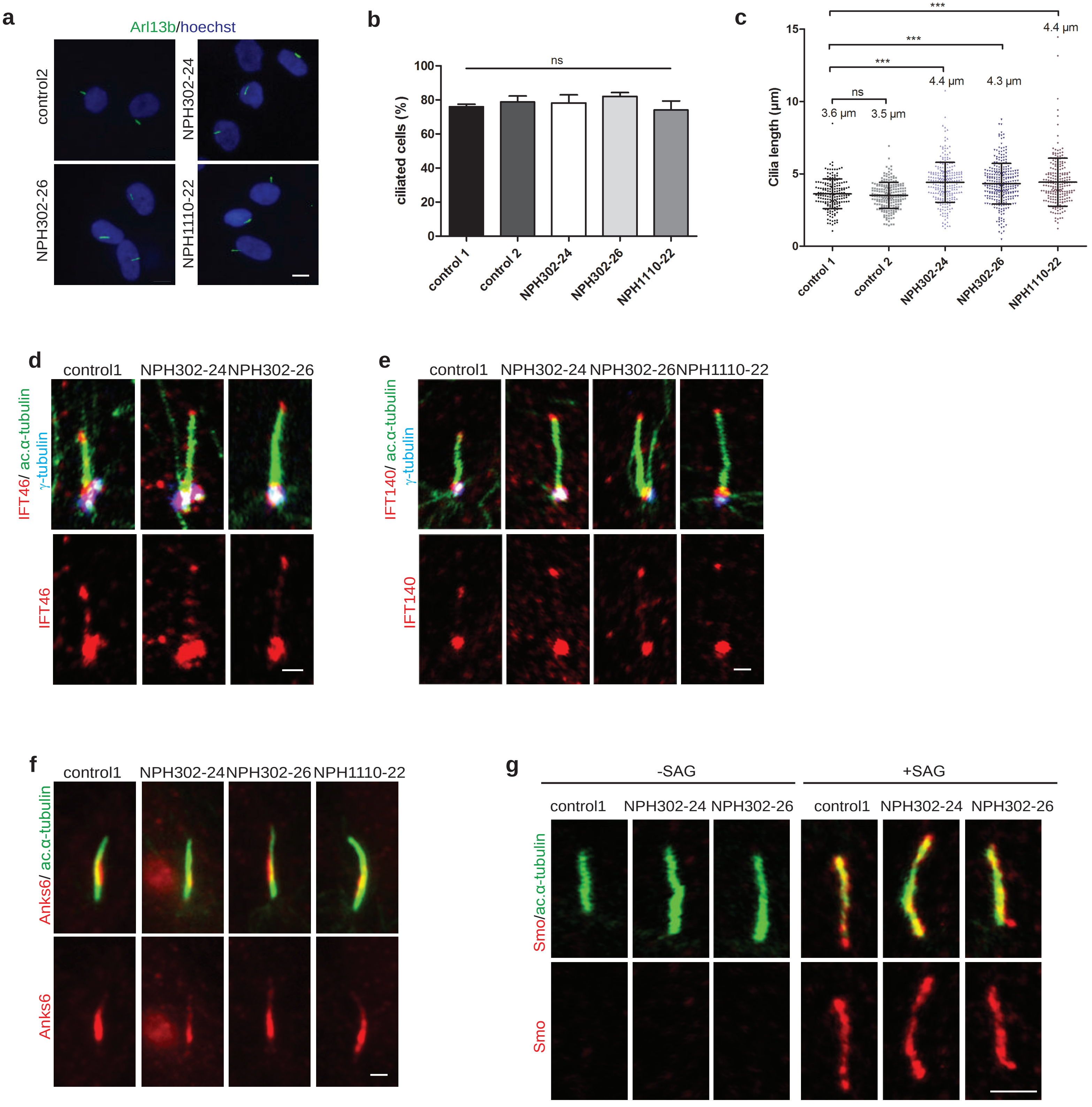

Supplementary Figure 6: Mutations in *TRAF3IP1* result in mild ciliary defects. (**a**) Ciliogenesis was analyzed by immunofluorescence in fibroblasts stained for ARL13B (cilia, green). Scale bar, 10µm. (**b**) Percentage of ciliated cells and (**c**) cilia length (n>200 cells from 4 independent experiments; ns: not-significant, ***P<0.001, Dunn's post-hoc test. Mean is indicated). (**d-f**) Ciliated fibroblasts from control and indicated affected individuals were stained for acetylated-α–tubulin (green, cilia) and γ-tubulin (light blue, basal body) and either the IFT-B component, IFT46 (red, **d**), the IFT-A subunit, IFT140 (red, **e**) or the Inversin marker, Anks6 (red, **f**). Scale bars, 1µm. (**g**) Ciliated fibroblasts from control and affected individuals treated (+) or not (-) with 100nM of the Hh agonist (SAG) for 24hrs were fixed and stained for acetylated-α-tubulin (green, cilia) and Smoothened (red). Scale bars, 1µm.

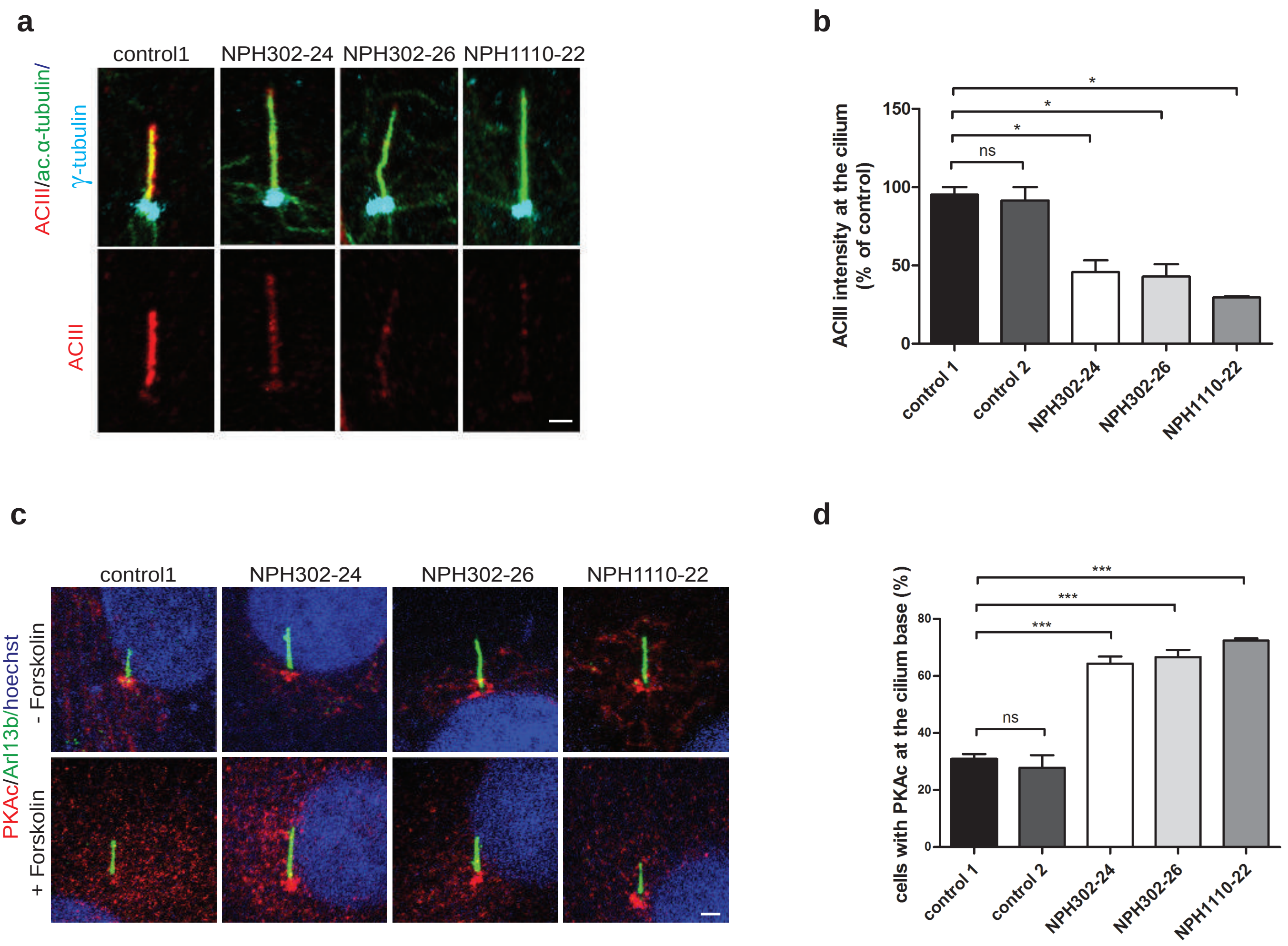

Supplementary Figure 7: Mutations in *TRAF3IP1* impair cAMP/PKA ciliary signaling. (**a**) ACIII (red), acetylated α-tubulin (green) and γ-tubulin (light blue) stainings in serum-starved fibroblasts. Scale bar, 1µm. (**b**) Intensity of ACIII within cilia (mean +/- SEM of n≥3 experiments (i.e. ≈100 cilia), *P<0.05, Dunn's post-hoc test). (**c**) Fibroblasts were treated with 1µM forskolin, an activator of ACs, for 1hr and stained for Arl13b (green) and PKAc (red). Scale bar, 2µm. (**d**) Percentage of cells with PKAc at the cilia base after forskolin treatment (mean +/- SEM of n=3 experiments (i.e. ≈120 cilia), *** P<0.001, Dunnett's post-hoc test).

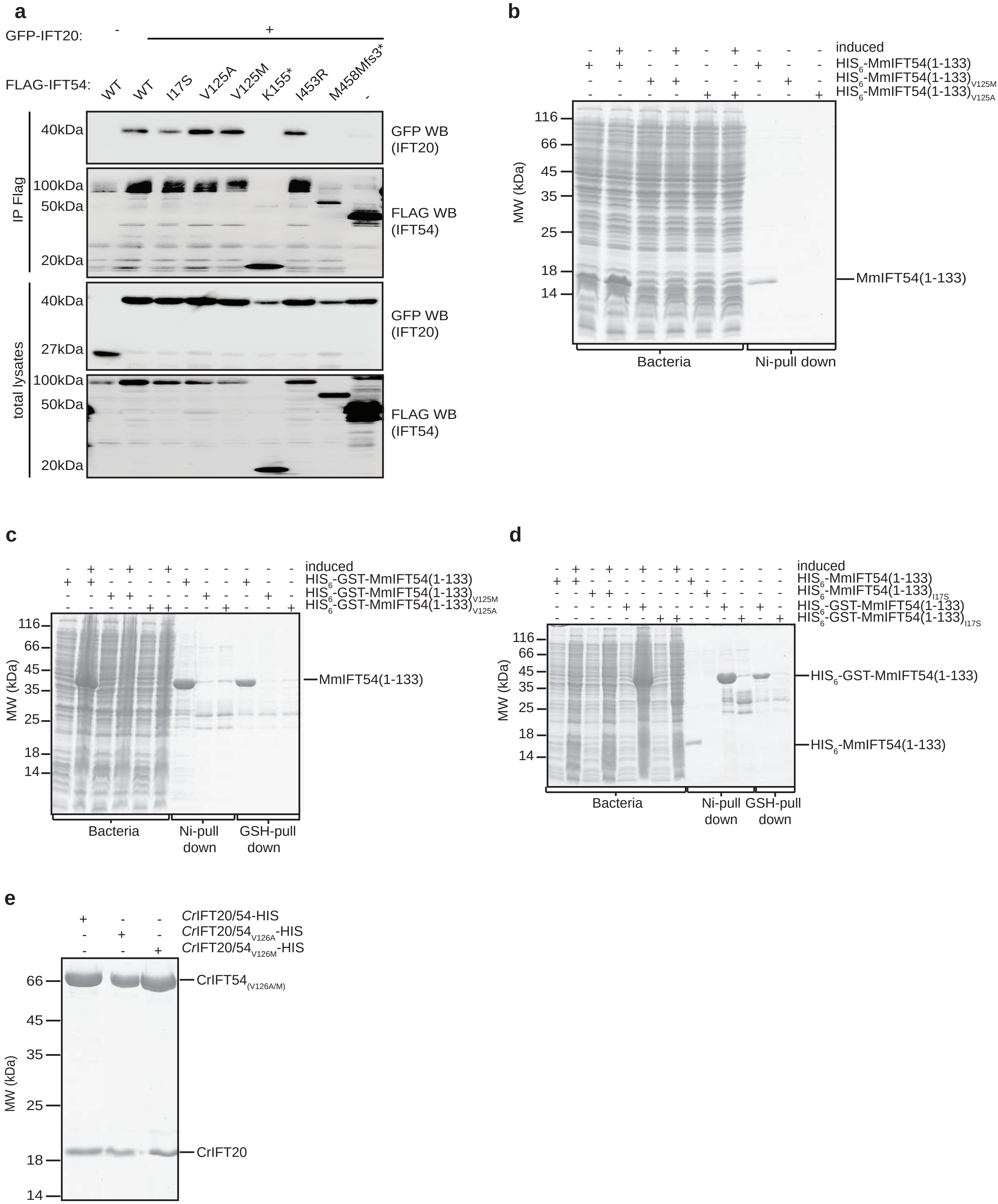

Supplementary Figure 8: Missense mutations of *TRAF3IP1* do not affect recruitment of IFT20 but affect protein solubility. (**a**) Lysates from HEK293T cells co-expressing Flag-tagged WT or mutant forms of *Mm*IFT54 (p.K155*, p.I453R and p.M458Mfs3* correspond to the human mutations p.R155*, p.M520R and p.M525Mfs3*) and IFT20-GFP (as indicated) were immunoprecipitated (IP) with an anti-Flag antibody. The co-immunoprecipitation of IFT20-GFP and Flag-IFT54 constructs was followed by Western-blot (WB) using GFP and Flag antibodies. (**b-d**) Expression tests in *E. coli* of WT and mutant *Mm*IFT54 N-terminal CH domain mutants p.V125M and p.V125A (**b-c**) or p.I17S (**d**); (Histidine (**b** and **d**) or GST (**c-d**) tagged forms). (**e**) Coomassie stained SDS gel of histidine-tagged forms of *Chlamydomonas reinhardtii* (*Cr*) IFT54 WT, V126A and V126M mutants in complex with IFT20 after size exclusion chromatography.

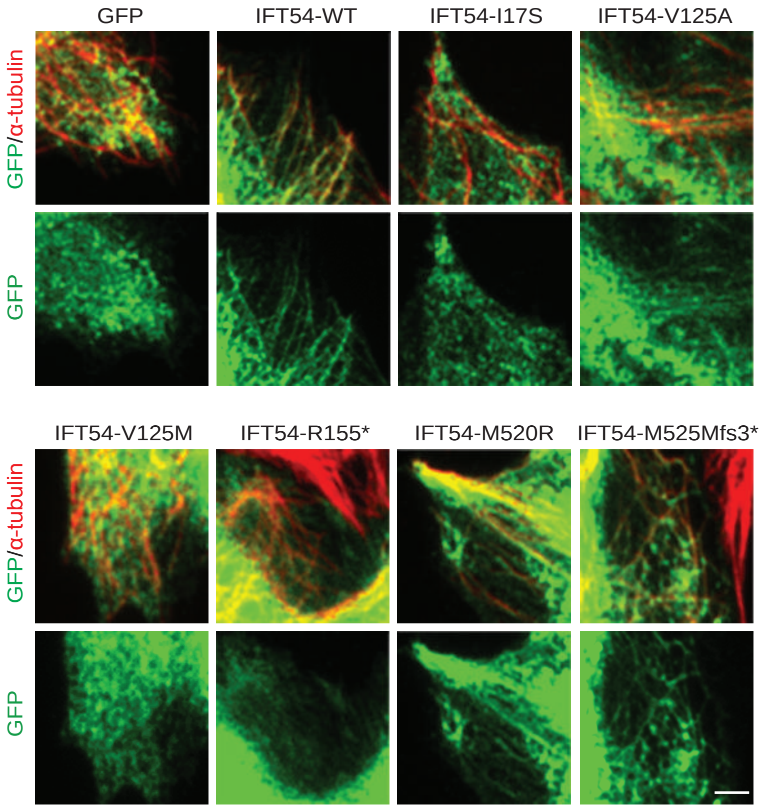

Supplementary Figure 9: *TRAF3IP1* mutations impair IFT54 localization along the cytoplasmic microtubules. RPE1 cells transfected with WT or mutant forms of GFP-IFT54 (green) were treated with 1μM taxol for 1hr to stabilize the microtubule network then fixed with MeOH and stained α-tubulin (red). Scale bars, 2μm.

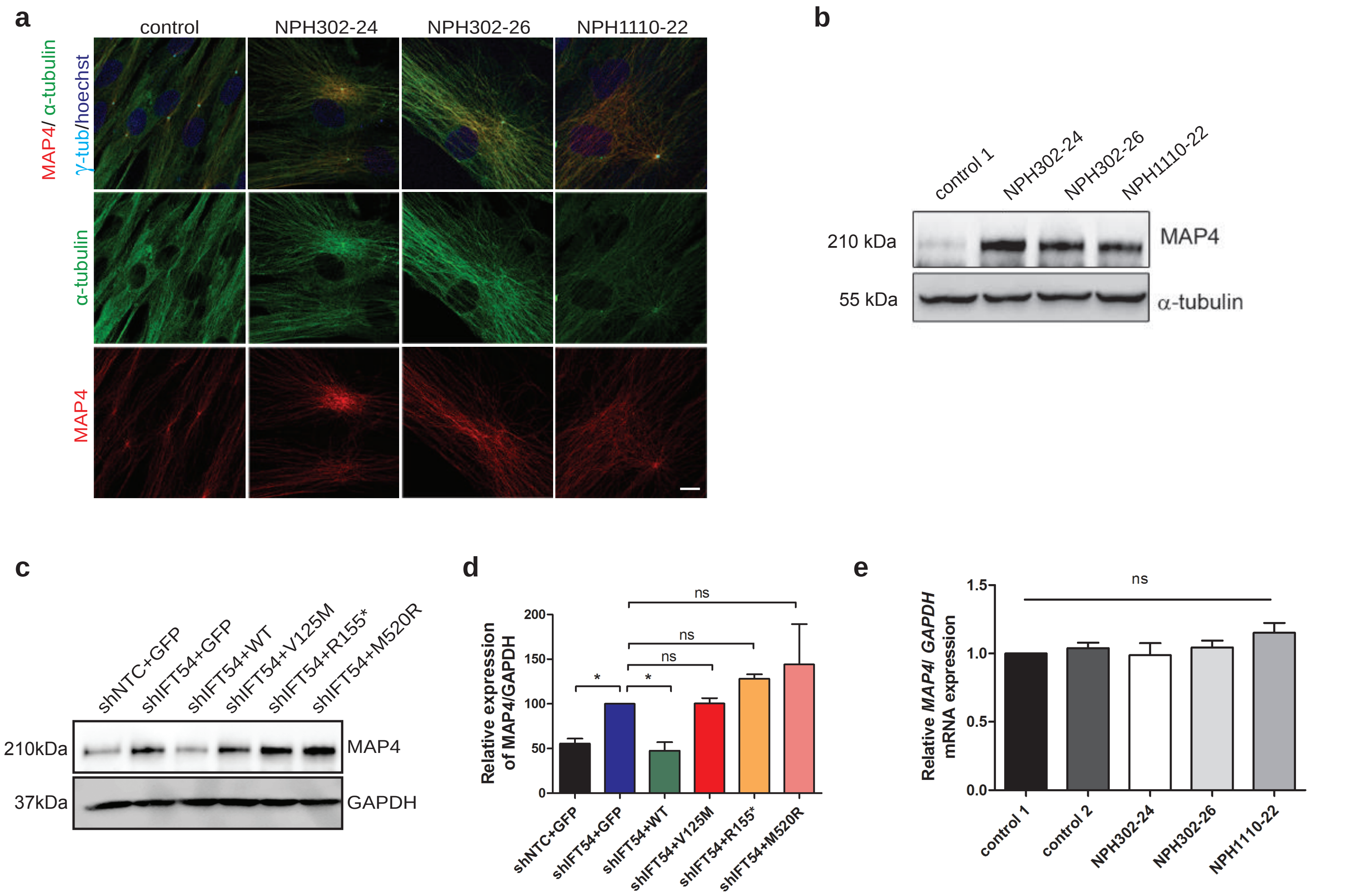

Supplementary Figure 10: *TRAF3IP1* mutations impair MAP4 localization and expression. (**a**) Fibroblasts from control and affected individuals were fixed in MeOH and stained for α-tubulin (green, microtubules) and MAP4 (red). Scale bars, 10µm. (**b**) Western blot of MAP4 and α-tubulin (indicating that the same amount of cytoskeletal material was extracted) in fibroblasts from control and affected individuals. (**c**) Expression of MAP4 and GAPDH (loading control) in control and *Traf3ip1*-KD mIMCD3 cells expressing GFP or GFP-IFT54 fusions were analyzed by Western blot. (**d**) Relative expression of MAP4 normalized to that of GAPDH analyzed by WB as in (**c**) (mean +/-SEM of n=3 independent experiments, *p<0.05, one sample *t*-test, ns: not significant) (**e**) Relative expression of MAP4 normalized to that of GAPDH was analyzed by qPCR in control and mutant fibroblasts.

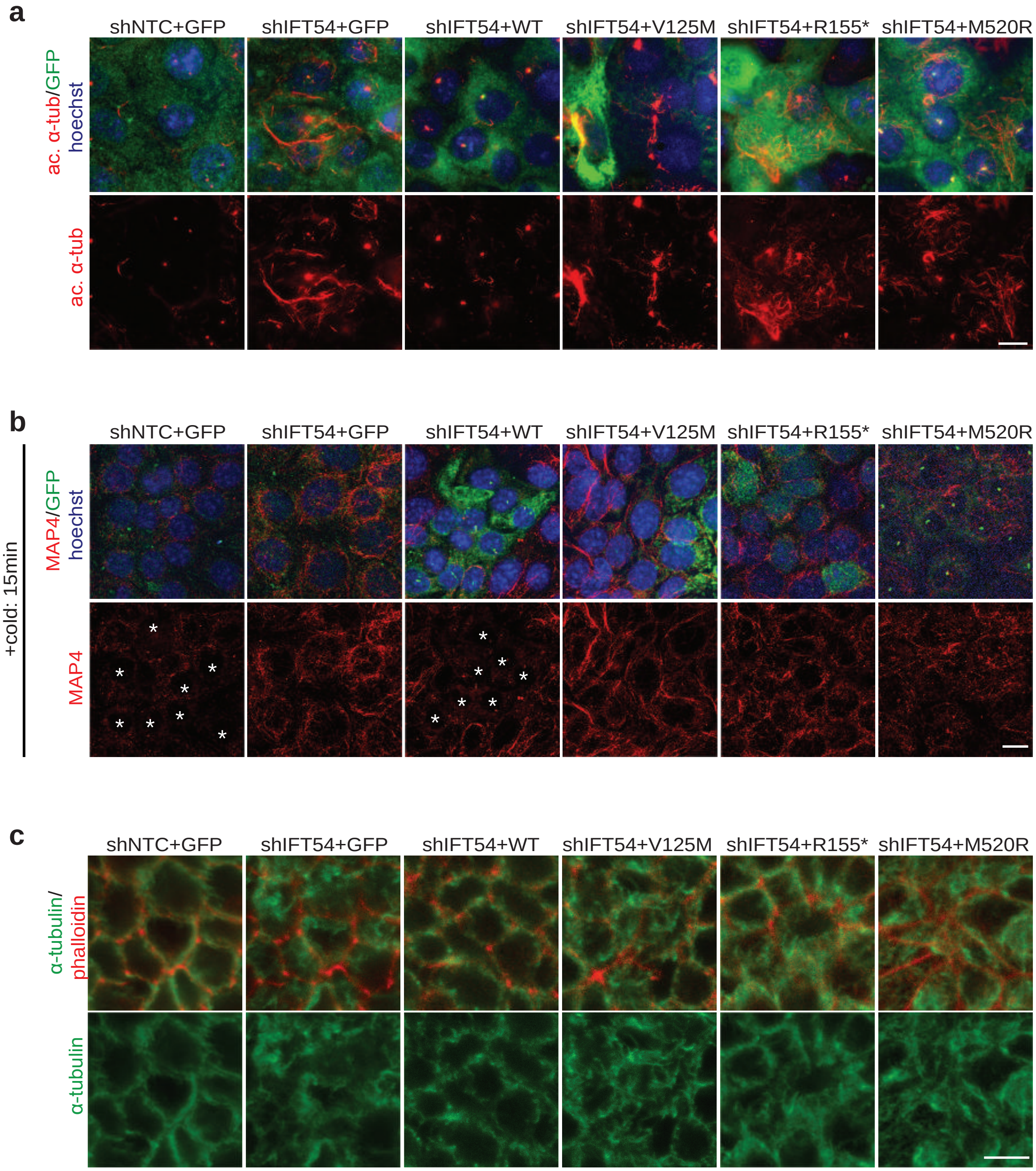

Supplementary Figure 11: *TRAF3IP1* mutations impair microtubule stability and organization. (**a-b**) Control and *Traf3ip1*-KD mIMCD3 cells expressing either GFP or IFT54 GFP-fusions (green) were either fixed directly in MetOH and stained for acetylated α–tubulin (red, **a**), or treated 15 min on ice before MetOH fixation and stained for MAP4 (red, **b**) Asterisks in (**b**) point to cells with no remaining MAP4 staining after cold treatment. Scale bars, 10µm. (**c**) Control and *Traf3ip1*-KD mIMCD3 cells expressing either GFP or IFT54 GFP-fusions grown on filters were fixed and stained for α-tubulin (green, microtubules) and for actin using phalloidin (red). Confocal images corresponding to representative x,y mid-sections are shown. Scale bars, 10µm.

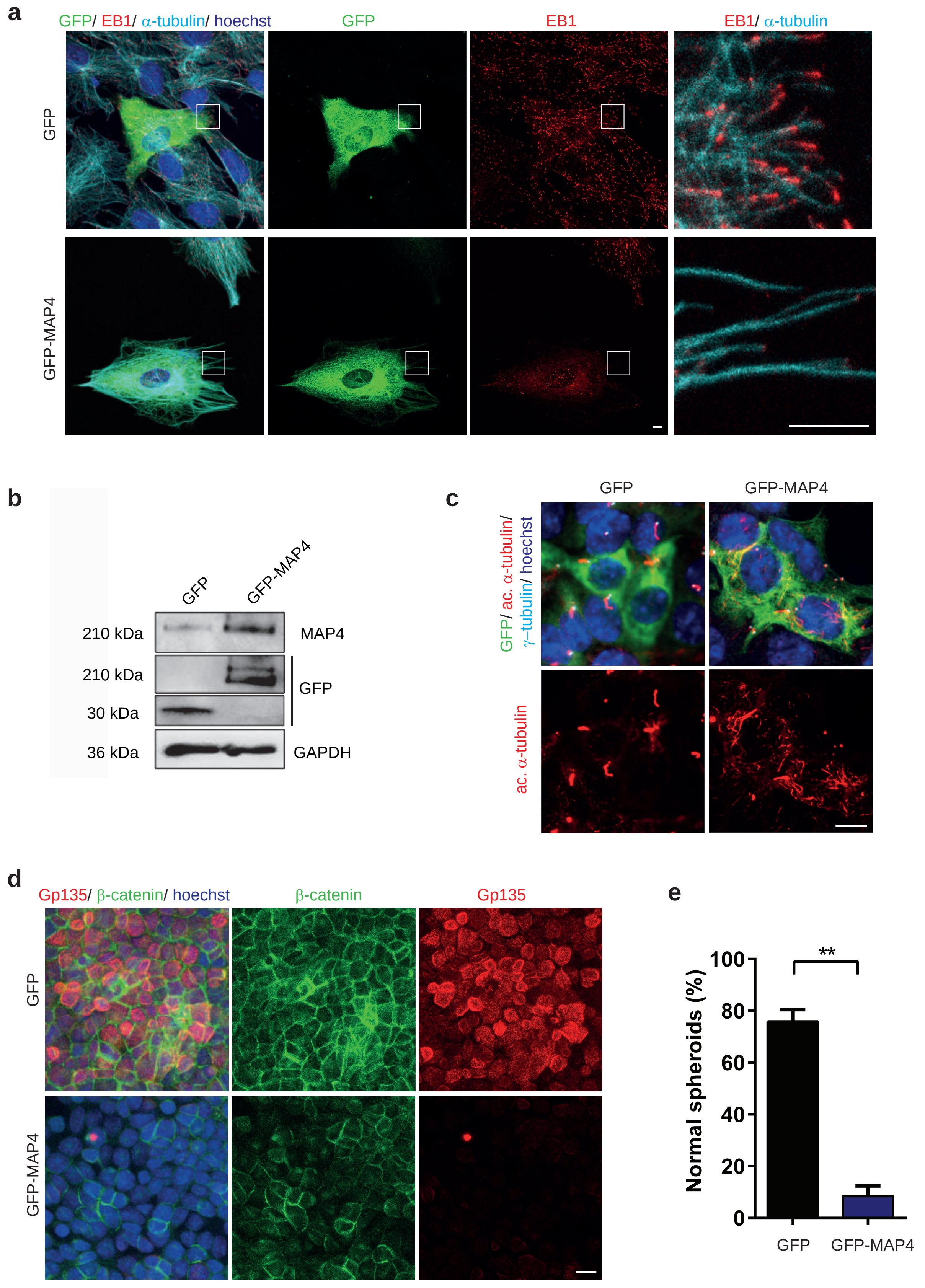

Supplementary Figure 12: MAP4 overexpression recapitulates *Traf3ip1*-KD phenotypes. (**a**) Control fibroblasts transfected with GFP or GFP-MAP4 were fixed with MeOH and stained for GFP (green), EB1 (red) and α-tubulin (light blue). Enlarged regions highlight EB1 at the microtubule plus-tips. Scale bar, 5µm. (**b**) Expression of MAP4, GFP and GAPDH (loading control) in mIMCD3 cells stably expressing GFP or GFP-MAP4 were analyzed by western blot. (**c**) mIMCD3 cells expressing GFP or GFP-MAP4 were fixed with MeOH and stained for GFP (green), aceylated α-tubulin (red, stable microtubules) and γ-tubulin (light blue). Scale bar, 10µm. (**d**) mIMCD3 cells expressing GFP or GFP-MAP4 grown on filters were fixed 6hrs after Ca2+ switch and stained for Gp135 (red, apical membrane marker) and β-catenin (green, adherens junctions). Scale bar, 10µm. (**e**) Percentage of normal spheroids with lumen of mIMCD3 cells expressing either GFP or GFP-MAP4 grown on Matrigel for 5 days (mean +/- SD, n≥ 60 spheroids from 2 independent experiments, **p = 0.004, paired *t*-test).

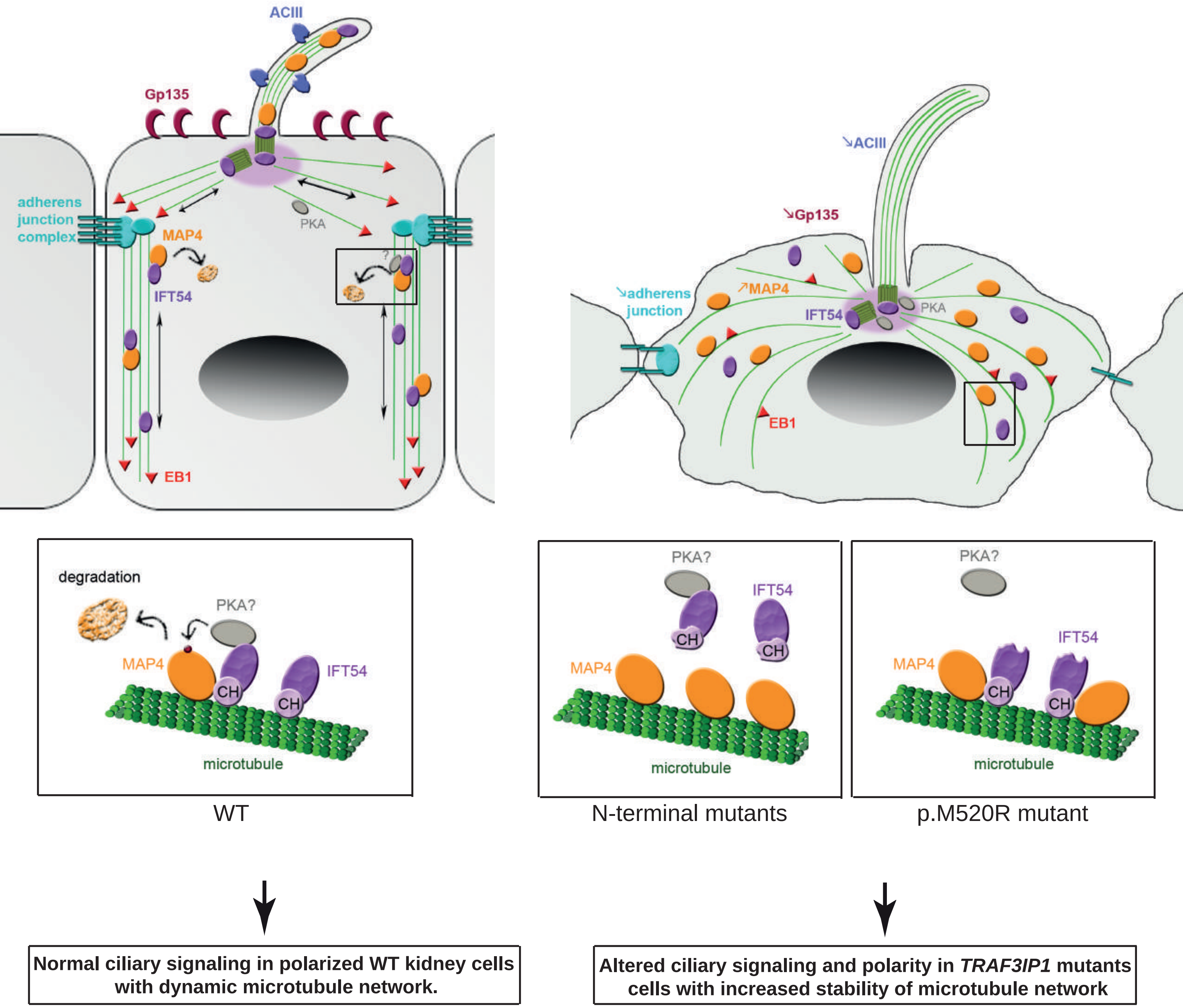

Supplementary Fig. 13: Model of the mechanisms by which *TRAF3IP1* mutations lead to epithelial degeneration. In WT cells, IFT54 enters the cilia together with MAP4 and ACIII, allowing proper ciliary signaling. In the cytoplasm, IFT54 may compete with MAP4 for binding to microtubules and/ or facilitates its phosphorylation by PKA, resulting in MAP4 dissociation from microtubules and likely MAP4 degradation (inset). Thereby, IFT54 regulates dynamic microtubules (with EB1 at their plus-ends) and correct microtubule network organization during epithelialization. Microtubules target adherens junctions and membrane proteins (e.g. Gp135) to apico or basolateral domains of the plasma membrane, establishing cell polarity. In mutant cells, in contrast, IFT54 cannot enter the cilia, resulting in decreased ciliary ACIII and MAP4 localization, associated with longer cilia and altered cAMP ciliary signaling. In the cytoplasm, N-terminal IFT54 mutants no longer bind to microtubules, increasing the number of microtubule-free sites for MAP4 binding. Conversely, the p.M520R mutant is still able to bind to MAP4 and to microtubules but it may impair MAP4 phosphorylation by PKA (insets). Both N-terminal and C-terminal mutations therefore result in a dramatic increase of MAP4 expression, leading to an over-stabilized and disorganized microtubule network which finally results in cellular polarity defects.

Figure 4b

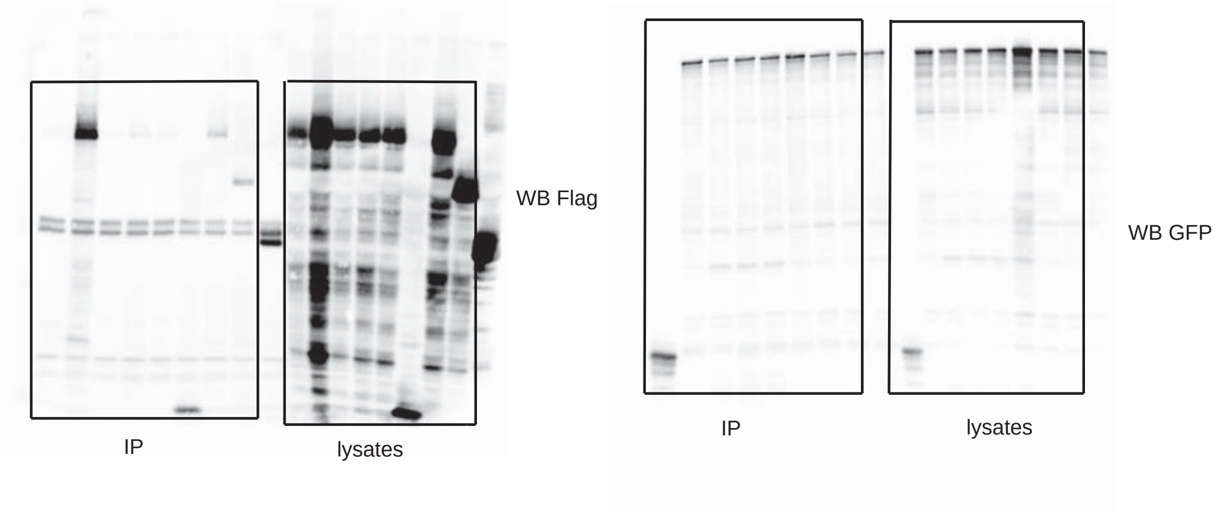

Figure 5a

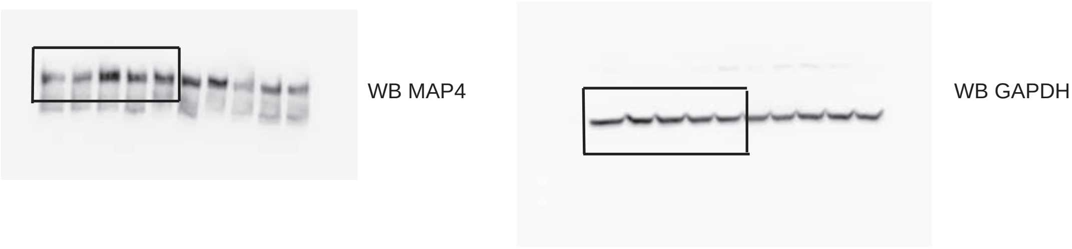

Figure 6d

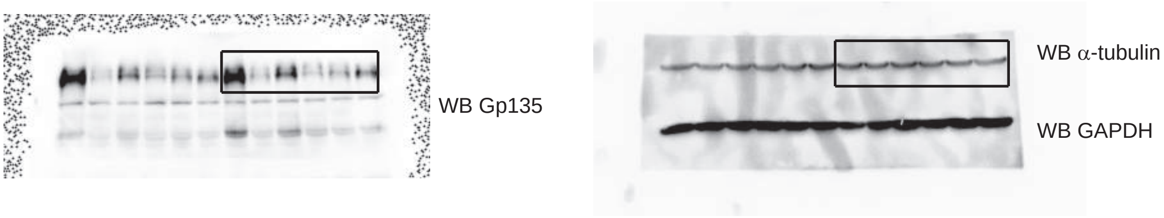

Supplementary Fig. 14: Full blots of the cropped images presented in the main text. The blot of Fig 6d was probed first with GAPDH (but there was a bubble) and then reprobe with α-tubulin.

	NPH1110-22	NPH579-22	NPH638-21	NPH302-23	A4336-22
Next generation sequencing	Ciliome			Whole exome	
Total variants called	6105 (1265)	5537 (1188)	7554 (1413)	79372 (28128)	205798
Amino Acid change; Unknown SNP variants (dbSNP132 and 1KG, MAF<1%)	212 (116)	216 (113)	280 (150)	1844 (1399)	1696
Rare variants (in-house database)	76 (67)	79 (68)	142 (117)	1192 (1039)	/
Linkage analysis	/	/	/	13 (12)[a]	160[a]
Quality filtering ; Compound heterozygote or homozygote variants (recessive disease model)	3 (2)	5 (4)	13 (7)	2 (2)	42
Surviving variants to evaluation	1 (1)	2 (2)	2 (1)	2 (2)	8 (8)

[a] linkage analysis (**Supplementary Fig. 1f-g**)

Supplementary table 1: Candidate gene selection from whole exome or ciliome resequencing in affected individuals. The number of variants and genes (in brackets) that remained after filtering strategy, are shown for each individual. Different filters were applied to exclude all variants located in non-exonic regions, pseudogenes, UTRs (...) or known polymorphic variants with a frequency above 1%, i.e. present in databases such as dbSNP, 1000 genomes (1KG) and all variants identified by in-house exome sequencing (less than 25 projects on 861 projects total). A quality filter was applied for all variants : all calls with a read coverage ≤ 2 and a Phred-scaled SNP quality of ≤ 20 were filtered out. Direct inspection was used to eliminate mismatches, incorrect locuses and bad ends.

	genomic position	Gene	RefSeq accession	Nt change c.	AA change p.	PP2	Sift	zygosity	Coverage ref/var	In house Data Base	Variation	ExAC (freq)	Sanger	Segregation
NPH1110-22	**chr2:239261597**	**TRAF3IP1**	**NM_015134**	**c.1575+6T>G**	**p./**	-	-	**hom**	**0/118**	**no**		**NA**	**yes**	**yes**
	**chr17:17136277**	**MPRIP**	**NM_015134**	**c.563_568del**	**p.Ser188_Ser189del[a]**	-	-	**het**	**19/7**	**no**	**rs202138172**	**0.0007939**	-	-
	**chr17:17126802**	**MPRIP**	**NM_015134**	**c.368C>T**	**p.T123M**	**0.998**	**0.16**	**het**	**7/11**	**yes**	**rs144748621**	**0.0002886**	-	-
	chr1:217774512	SPATA17	NM_138796	c.698C>T	p.P233L	0.689	0	het	63/27	yes	rs141925976	NA	-	-
	chr2:196738329	DNAH7	NM_018897	c.6376A>G	p.I2126V	0.86	0	het	47/31	no	no	-	-	-
	chr3:138525489	CEP70	NM_024491	c.944+1G>C	-	-	-	het	24/22	yes	rs139687358	NA	-	-
	chr4:5624344	EVC2	NM_147127	c.2421G>T	p.R807S	0.928	0.01	het	50/36	no	no	-	-	-
	chr5:75902024	IQGAP2	NM_006633	c.1253C>A	p.S418Y	0.249	0.03	het	36/36	yes	no	-	-	-
	chr6:151548482	CCDC170	NM_025059	c.767T>G	p.L256R	0.996	0.07	het	62/58	yes	rs192947987	0.000798722	-	-
	chr9:71750132	TMEM2	NM_001135820	c.242A>C	p.N81T	0.766	0.04	het	50/36	no	rs199978896	NA	-	-
	chr11:117373818	CEP164	NM_014956	c.1220C>T	p.S407F	0.842	0.04	het	30/22	yes	rs150314805	NA	-	-
	chr11:59559626	STX3	NM_001178040	c.404A>G	p.N135S	0.995	0.01	het	90/67	no	no	-	-	-
	chr16:672958	RHOT2	NM_138769	c.1558C>T	p.L520F	0.978	0.07	het	29/34	yes	rs55977091	0.000998403	-	-
	chr16:70969896	HYDIN	NM_001270974	c.7117G>C	p.E2373Q	0.862	0.22	het	183/59	yes	no	-	-	-
	chr17:7852450	CNTROB	NM_001037144	c.2627G>A	p.R876H	0.997	0	het	20/20	no	no	-	-	-
	chr18:19424154	MIB1	NM_020774	c.2151G>C	p.Q717H	0.858	0	het	34/21	no	no	-	-	-
	chr19:3981985	EEF2	NM_001961	c.857C>T	p.P286L	0.017	0.04	het	10/4	no	no	-	-	-
	chr20:62338349	ARFRP1	NM_001267548	c.93+2T>C	-	-	-	het	64/34	no	no	-	-	-
	chr22:30899735	SEC14L4	NM_174977	c.59G>A	p.R20Q	0.176	0.02	het	46/26	yes	no	-	-	-
NPH579-22	**chr2:239261575**	**TRAF3IP1**	**NM_015650.3**	**c.1559T>G**	**p.M520R**	**0.216**	**0.01**	**hom**	**01/58**	**no**	**no**	-	**yes**	**yes**
	**chr1:154251068**	**UBAP2L**	**NM_014847.3**	**c.1241T>C**	**p.V414A**	**0.294**	**0.01**	**hom**	**0/106**	**yes**	**rs149365430**	**0,00159744**	-	-
	**chr9:135781442**	**TSC1**	**NM_001162426.1**	**c.1523A>G**	**p.Y508C**	**0.02**	**0.02**	**het**	**30/14**	**yes**	**no**	-	-	-
	**chr9:135781443**	**TSC1**	**NM_001162426.1**	**c.1522T>C**	**p.Y508H**	**0.024**	**0.17**	**het**	**26/15**	**no**	**no**	-	-	-
	**chr16:30773903**	**RNF40**	**NM_014771.3**	**c.37G>C**	**p.G13R**	**0.006**	**0.08**	**hom**	**01/04**	**yes**	**no**	-	-	-
	chr1:228494987	OBSCN	NM_001271223.2	c.15092T>C	p.L5031P	0.998	0	het	11/6	no	no	-	-	-
	chr3:170843819	TNIK	NM_015028.2	c.1895G>A	p.R632H	0.337	0.02	het	38/46	no	no	-	-	-
	chr4:39277126	WDR19	NM_025132.3	c.3823C>G	p.P1275A	1	0	het	83/56	yes	rs200670161	0,000599042	-	-
	chr6:43524615	XPO5	NM_020750.2	c.3333A>G	p.I1111M	0.075	0.01	het	36/21	yes	rs112085617	0,000599042	-	-
	chr7:47384422	TNS3	NM_022748.11	c.2581C>T	p.R861C	0.241	0.02	het	16/19	no	no	-	-	-
	chr8:67923029	PPP1R42	NM_001013626.2	c.473T>C	p.I158T	0.936	0	het	33/32	no	no	-	-	-
	chr10:50009617	AGAP6	NM_001077665.2	c.1492A>G	p.M498V	1	0.06	het	176/70	yes	rs202194597	0,000199681	-	-
	chr11:66299202	BBS1	NM_024649.4	c.1684G>T	p.D562Y	0.999	0.01	het	63/50	no	no	-	-	-
	chr12:112616868	HECTD4	NM_001109662.3	c.10792G>A	p.V3598M	0.074	0	het	11/5	no	no	-	-	-
	chr12:121949060	WDR66	NM_144668.5	c.1268G>T	p.W423L	0.411	0.01	het	34/24	yes	rs151062299	0,00179712	-	-
	chr14:90689568	TTC7B	NM_001010854.1	c.922C>T	p.R308W	0.991	0.11	het	27/29	yes	rs143808424	NA	-	-
	chr19:32879317	CEP89	NM_032816.3	c.2197C>T	p.R733*	-	-	het	44/27	no	rs146991222	NA	-	-
NPH638-21	**chr2:239237346**	**TRAF3IP1**	**NM_015650.3**	**c.374T>C**	**p.V125A**	**0.799**	**0**	**het**	**74/77**	**no**	**no**	-	**yes**	**yes**
	**chr2:239237435**	**TRAF3IP1**	**NM_015650.3**	**c.463C>T**	**p.R155***	-	-	**het**	**69/61**	**no**	**no**	-	**yes**	**yes**
	**chr2:130897506**	**CCDC74B**	**NM_207310.2**	**c.965C>T**	**p.T322M**	**0.002**	**0.54**	**het**	**76/37**	**no**	**no**	-	-	-
	**chr2:130898815**	**CCDC74B**	**NM_207310.2**	**c.599T>A**	**p.V200D**	**0.589**	**0.01**	**het**	**199/21**	**yes**	**no**	-	-	-
	**chr4:56017835**	**CEP135**	**NM_025009.4**	**c.2990C>T**	**p.S997L**	**0.076**	**0.01**	**het**	**31/24**	**yes**	**rs146076380**	**0.00319489**	-	-
	**chr4:56020725**	**CEP135**	**NM_025009.4**	**c.3265T>A**	**p.L1089I**	**0.803**	**0.01**	**het**	**94/92**	**yes**	**rs76659072**	**0.00259585**	-	-
	**chr5:90685822**	**GPR98**	**NM_032119.3**	**c.6317C>T**	**p.A2106V**	**0.997**	**0.06**	**het**	**64/57**	**yes**	**rs186999408**	**0.00179712**	-	-
	**chr5:90791036**	**GPR98**	**NM_032119.3**	**c.14207C>G**	**p.S4736C**	**0.63**	**0.02**	**het**	**120/99**	**no**	**rs201478715**	**0.000399361**	-	-
	**chr8:85113091**	**LRRCC1**	**NM_033402.4**	**c.536G>A**	**p.R179Q**	**0.024**	**0.42**	**het**	**33/34**	**yes**	**rs76773098**	**0.00379393**	-	-
	**chr8:85126746**	**LRRCC1**	**NM_033402.4**	**c.1330G>A**	**p.E444K**	**0.547**	**0.18**	**het**	**60/52**	**yes**	**rs201242609**	**0.00319489**	-	-
	**chr9:120394636**	**CDK5RAP2**	**NM_018249.5**	**c.5454T>G**	**p.I1818M**	**0.356**	**0.27**	**hom**	**3/111**	**yes**	**rs114128928**	**0.00758786**	-	-
	**chr15:85741469**	**AKAP13**	**NM_006738.5**	**c.8044C>T**	**p.R2682W**	**0.95**	**0.11**	**het**	**15/22**	**yes**	**rs116249792**	**0.00738818**	-	-
	**chr15:86259124**	**AKAP13**	**NM_006738.5**	**c.5717C>G**	**p.S1906C**	**0.996**	**0.02**	**het**	**73/49**	**no**	**no**	-	-	-
	chr1:226994906	CDC42BPA	NM_003607.3	c.4945A>G	p.M1649V	0.922	0.05	het	81/55	yes	rs145538584	0.00459265	-	-
	chr10:112764379	SHOC2	NM_007373.3	c.988C>T	p.L330F	0.081	0.01	het	29/22	no	no	-	-	-
	chr12:110884101	CCDC63	NM_152591.1	c.925C>T	p.R309W	0.999	0	het	57/53	yes	rs116780136	0.00239617	-	-
	chr12:53715216	CALCOCO1	NM_020898.2	c.1370A>G	p.E457G	0.994	0	het	32/31	yes	rs34311341	0.00379393	-	-
	chr14:50772425	NIN	NM_020921.3	c.857T>C	p.M286T	0.772	0.03	het	124/106	yes	rs147260112	0.000599042	-	-
	chr15:101048653	LRRK1	NM_024652.3	c.3295C>T	p.L1099F	0.998	0.12	het	62/46	yes	rs200404538	0.000399361	-	-
	chr16:23700582	PLK1	NM_005030.3	c.1294G>A	p.V432M	0.881	0.06	het	71/58	no	no	-	-	-
	chr16:66734304	DYNC1LI2	NM_006141.2	c.707C>T	p.A236V	0.93	0.36	het	57/42	yes	rs138998809	0.00119808	-	-
	chr16:84155636	DNAAF1	NM_178452.4	c.628G>A	p.V210M	0.75	0.01	het	103/79	yes	rs76598454	0.000599042	-	-
	chr17:9598268	WDR16	NM_145054.4	c.571A>G	p.N191D	0.842	0.01	het	20/15	no	no	-	-	-
	chr2:217814989	TNS1	NM_022648.4	c.4340C>T	p.P1447L	0.094	0.01	het	106/75	yes	rs144860480	0.000199681	-	-
	chr2:218738089	TTLL4	NM_014640.4	c.413C>T	p.S138L	0.798	0.57	het	138/81	yes	rs140587312	0.00159744	-	-
	chr2:227906255	DAW1	NM_178821.1	c.775G>A	p.G259S	1	0.06	het	97/80	yes	rs35027781	0.00519169	-	-
	chr2:47051789	TTC7A	NM_020458.2	c.2061G>C	p.E687D	0.221	0.02	het	07/12	yes	rs147410586	0.00139776	-	-
	chr3:121694548	GOLGB1	NM_001256486.1	c.5975G>A	p.R1992Q	0.996	0	het	124/113	yes	rs111753163	0.00399361	-	-
	chr3:47411082	PTPN23	NM_015466.2	c.3284G>C	p.G1095A	0.772	0.47	het	02/09	yes	rs138127717	0.000199681	-	-
	chr4:126240270	FAT4	NM_024582.4	c.2704_2706del	p.V903del	-	-	het	108/77	yes	no	-	-	-
	chr5:10618515	ANKRD33B	NM_001164440.1	c.437T>C	p.V146A	0.471	0.01	het	19/06	no	no	-	-	-
	chr9:117168826	DFNB31	NM_001173425.1	c.2045G>C	p.R682P	0.996	0	het	13/08	no	no	-	-	-
NPH302-23	**chr2:239237345**	**TRAF3IP1**	**NM_015650.3**	**c.373G>A**	**p.V125M**	**1**	**0.01**	**hom**	**16/03**	**no**	**no**	-	**yes**	**yes**
	**chr2:241073350**	**MYEOV2**	**NM_138336.1**	**c.229G>A**	**p.D77N**	**0.803**	**0.03**	**hom**	**01/13**	**no**	**no**	-	**yes**	**yes**
	chr2:238277479	COL6A3	NM_057167.3	c.4009C>A	p.H1337N	0.959	0	het	10/14	yes	no	-	-	-
	chr2:238428661	MLPH	NM_024101.6	c.665delA	p.D222Afs*102	-	-	het	08/05	no	no	-	-	-
A4336-22	**chr2:239229353**	**TRAF3IP1**	**NM_015650.3**	**c.50T>G**	**p.I17S**	**0.997**	**0**	**hom**	**0/6**	**no**	**no**	-	**yes**	**yes**
	**chr3:50251833**	**SLC38A3**	**NM_006841.4**	**c.103dup**	**p.V35Gfs*27**	-	-	**hom**	**0/13**	**NA**	**rs5848902**	**NA**	-	-
	**chr5:6652043**	**SRD5A1**	**NM_001047.2**	**c.382G>A**	**p.G128S**	**0.937**	**0**	**hom**	**0/80**	**NA**	**rs140569241**	**0.001095**	-	-
	**chr12:52215083**	**FIGNL2**	**NM_001013690**	**c.1115C>A**	**p.P372H**	**0.466**	**NA**	**hom**	**0/2**	**NA**	**no**	-	-	-
	**chr12:93100466**	**C12orf74**	**NM_001037671**	**c.59C>G**	**p.P20R**	**0.939**	**0**	**hom**	**0/11**	**NA**	**rs142446097**	**0.001139**	-	-
	**chr16:53679577**	**RPGRIP1L**	**NM_015272.2**	**c.2643T>A**	**p.N881K**	**0.02**	**0.05**	**hom**	**01/77**	**NA**	**rs139503476**	**0.0005652**	-	-
	**chr16:53720481**	**RPGRIP1L**	**NM_015272.2**	**c.640G>A**	**p.V214I**	**0.004**	**0.14**	**hom**	**0/105**	**NA**	**rs139067427**	**0.0005292**	-	-
	**chr17:73916166**	**FBF1**	**NM_001080542**	**c.1808G>A**	**p.R603Q**	**0.999**	**0**	**hom**	**0/14**	**NA**	**no**	-	-	-
	**chr17:73922167**	**FBF1**	**NM_001080542**	**c.770C>G**	**p.P257R**	**0.935**	**0**	**hom**	**01/13**	**NA**	**rs201197761**	**NA**	-	-
	**chr19:2247841**	**SF3A2**	**NM_007165.4**	**c.691A>G**	**p.K231E**	**0.588**	**0.48**	**hom**	**0/2**	**NA**	**no**	-	-	-
	**chr19:52448488**	**ZNF613**	**NM_001031721**	**c.1244T>C**	**p.L415P**	**1**	**0**	**hom**	**0/14**	**NA**	**no**	-	-	-

Supplementary table 2 : Overview of Candidate Genes from Ciliome or Exome Data in Affected Individuals. Genes written in bold letters indicate variants that are compatible with a recessive model. Segregation analysis revealed that *TRAF3IP1* variants segregate with disease in these families (shown in the pedigree in Supplementary Fig.1a-e). Columns from left to right: position of the variant in hg19 (genomic position), chromosome (chr), name of the gene in which the variant is detected (Gene), RefSeq accession, Nucleotide (Nt) and Amino Acid (AA) change, Polyphen 2 (PP2) and SIFT Score (red : predict disease causing, green: predict benign), homozygotes (hom) or heterozygotes (het) Variants, number of reads whereby position is covered for reference allele (Ref) and variant allele (Var), Variants found in house Data Base, variations ID in dbSNP (variation), Allelic frequency in Exome Aggregation Consortium (ExAC), Validation by Sanger sequencing (Sanger), variant segregate with disease (Segregation).

[a] The serine stretch mutated in affected individual NPH1110-22 is not conserved throughout evolution (from analysis with Clustal Omega)

name	purpose	species	position on cDNA	exon	sequence
TRAF3IP1-1F	gene screening	human		1	GCACTGTGGGATGGAAACCG
TRAF3IP1-1R	gene screening	human		1	CAGAAGCAGCTCTGCCAGCAAT
TRAF3IP1-2F	gene screening	human		2	GTGGATGAGGCTGATGAGG
TRAF3IP1-2R	gene screening	human		2	CCAGTTGCCACAATGAGAAA
TRAF3IP1-3F	gene screening	human		3	TGTGGCAACTGGATGTCAT
TRAF3IP1-3R	gene screening	human		3	GGGTTCCCGACTTTCTACTG
TRAF3IP1-4F	gene screening	human		4	GGGTGGACGCTACTGTTA
TRAF3IP1-4R	gene screening	human		4	AATTCTTCCTTCTGTTTTCGAT
TRAF3IP1-5F	gene screening	human		5	TTAGCTGGAGAGAAGGGAG
TRAF3IP1-5R	gene screening	human		5	TAGGCATAAAAGAAAACCAGTT
TRAF3IP1-6F	gene screening	human		6	TTATAGAAAATATCTTGGCATA
TRAF3IP1-6R	gene screening	human		6	CCCACAGAAAATCAGAG
TRAF3IP1-7F	gene screening	human		7	GCTTGAAAAATAAACCTGCT
TRAF3IP1-7R	gene screening	human		7	CACGCATGTGTATGTAACAG
TRAF3IP1-8F	gene screening	human		8	AAATCCCAGCTAAAACAA
TRAF3IP1-8R	gene screening	human		8	AAGTTATGTCCAGTCTTCAATA
TRAF3IP1-9F	gene screening	human		9	ATGTTAATGAAGCCGCTGAT
TRAF3IP1-9R	gene screening	human		9	ATGCCACCTCCTCTCACTT
TRAF3IP1-10F	gene screening	human		10	TCCTGTTTCTATTTAGTACCAT
TRAF3IP1-10R	gene screening	human		10	AGAGTCCACATTCACATTC
TRAF3IP1-11F	gene screening	human		11	GGGAGCTGACATGTGAC
TRAF3IP1-11R	gene screening	human		11	GCACTAAATAAATAGCAGCAG
TRAF3IP1-12F	gene screening	human		12	ACTTTCTGATTGGTCGGGTTA
TRAF3IP1-12R	gene screening	human		12	TGTCTGTTGGCCTATGGTGT
TRAF3IP1-13F	gene screening	human		13	TTAGATTCCTCTCTGCCGAC
TRAF3IP1-13R	gene screening	human		13	GGGGGAAAAAGAAGATTCA
TRAF3IP1-14F	gene screening	human		14	TTTTGAGATCCACAGAAGCAT

TRAF3IP1-14R	gene screening	human		14	CAGTGTGACCCAGACCAG
TRAF3IP1-15F	gene screening	human		15	TAGGTTTTTGGGATAGAGAAT
TRAF3IP1-15R	gene screening	human		15	AAAGATGCTCCAACTTGTAA
TRAF3IP1-16F	gene screening	human		16	GCCCTGTTCTGCCTTTGGACT
TRAF3IP1-16R	gene screening	human		16	CCACCCCAACCTGTTCCTG
TRAF3IP1-17F	gene screening	human		17	AAGAAGCCAACACACAAT
TRAF3IP1-17R	gene screening	human		17	TAACTAGCCAGTATTCCATCT
TRAF3IP1h-7F	cDNA verification	human	7		GCGGCGGTGGTGAGGCGGAC
TRAF3IP1h-156R	cDNA verification	human	156		TCATCTCGGCGTCTGTGTAG
TRAF3IP1h-563F	cDNA verification	human	563		TGAAAGAAGACCGCAAGCCA
TRAF3IP1h-923F	cDNA verification	human	923		GCTCAGGGGAGATGTCTAAA
TRAF3IP1h-1410F	cDNA verification	human	1410		GGTCAAACGGCAAGACAGCA
TRAF3IP1h-1917F	cDNA verification	human	1917		AGACTGTGCCGTGGAGCCCT
TRAF3IP1m-24F	cDNA verification	mouse	24		GACGCAGGAGGCTCTGGGCA
TRAF3IP1m-292R	cDNA verification	mouse	292		GTTCTTTCAGGTTCGTGCCC
TRAF3IP1m-563F	cDNA verification	mouse	563		AAGACAGCAAGCCTCGGGAG
TRAF3IP1m-1022F	cDNA verification	mouse	1022		TAAAACCATCAAAACGGCGA
TRAF3IP1m-1410F	cDNA verification	mouse	1410		CGGTGGGCTCGTGAAGAAGA
Flag-IFT54m-mutV125A-F	mutagenesis	mouse			CAGTGATGAGGCTGCGAAGAGAGTCTTAGCTG
Flag-IFT54m-mutV125A-R	mutagenesis	mouse	V125A		CAGCTAAGACTCTCTTCGCAGCCTCATCACTG
Flag-IFT54m-mutV125M-F	mutagenesis	mouse			CTCCAGTGATGAGGCAATGAAGAGAGTCTTAG
Flag-IFT54m-mutV125M-R	mutagenesis	mouse	V125M		CTAAGACTCTCTTCATTGCCTCATCACTGGAG
Flag-IFT54m-mutK155*-F	mutagenesis	mouse			CAACAAGAGTGGGTAGGAGGAAGAGTCCAGAATAC
Flag-IFT54m-mutK155*-R	mutagenesis	mouse	K155*		GTATTCTGGACTCTTCCTCCTACCCACTCTTGTTG
Flag-IFT54m-mutM458Mfs3X-F	mutagenesis	mouse			GCAGACATTGACATGGTTAGGTGCCGTCAGGGGAGC
Flag-IFT54m-mutM458Mfs3X-R	mutagenesis	mouse	M458Mfs3X		GCTCCCCTGACGGCACCTAACCATGTCAATGTCTGC
Flag-IFT54m-mutI520R-F	mutagenesis	mouse	I520R		CTCAGCTGTCAGAACGCGCAGATATTGATATGGTGCCGTCAG

Flag-IFT54m-mutI520R-R	mutagenesis	mouse			CTGACGGCACCATATCAATATCTGCGCGTTCTGACAGCTGAG
GFP-IFT54-mutM520R-R	mutagenesis	human			CCATTTCAATTTCTGATCTTTCTGAGAGCTGAGG
GFP-IFT54-mutM520R-F	mutagenesis	human	M520R		CCTCAGCTCTCAGAAAGATCAGAAATTGAAATGG
GFP-IFT54-mutV125M-F	mutagenesis	human			AGTGACGATGCGATGCGGAGGGTTTTAG
GFP-IFT54-mutV125M-R	mutagenesis	human	V125M		TAAAACCCTCCGCATCGCATCGTCACTAG
GFP-IFT54-mutR155*-F	mutagenesis	human			AGAATGTGTGAGAAGAAGAGTCCAGAG
GFP-IFT54-mutR155*-R	mutagenesis	human	R155*		ACTCTGGACTCTTCTTCTCACACATTC
IFT54 h qPCR F	qPCR	human	116	1/2	TCACGGAGGTGATTAGAATGACT
IFT54 h qPCR R	qPCR	human	242	3	ACAACCACGTCTATGGCCTTT
MAP4 h qPCR F	qPCR	human	1665	7	AGCACCCCTGGCTAAGGAT
MAP4 h qPCR R	qPCR	human	1896	8	CCCCGTTCCTGTGATGGTTT
MAP4 m qPCR F	qPCR	mouse	2774	12/13	GCCGGGCCAAAGTAGAGAAAA
MAP4 m qPCR R	qPCR	mouse	2843	13	GTGACTGCATTAGGTTCAGGC
Traf3ip1 shRNA # 461	shRNA	mouse			CCGGATGAGCTGCTTCAATTGATTGCTCGAGCAATCAATTGAAGCAGCTCATTTTTTG
Traf3ip1 shRNA # 462	shRNA	mouse			CCGGCATCTAGGTCCTCGACGTTAACTCGAGTTAACGTCGAGGACCTAGATGTTTTTG
Traf3ip1 shRNA # 463	shRNA	mouse			CCGGGAATACACAAAGAGGATAAACCTCGAGGTTTATCCTCTTTGTGTATTCTTTTTG

Supplementary table 3: List of forward and reverse primers used to perform PCR investigations and sequencing, as well as mutagenesis, quantitative Real-Time PCR and shRNA.
